# Data-driven biomarkers better associate with stroke motor outcomes than theory-based biomarkers

**DOI:** 10.1093/braincomms/fcae254

**Published:** 2024-07-31

**Authors:** Emily R Olafson, Christoph Sperber, Keith W Jamison, Mark D Bowren, Aaron D Boes, Justin W Andrushko, Michael R Borich, Lara A Boyd, Jessica M Cassidy, Adriana B Conforto, Steven C Cramer, Adrienne N Dula, Fatemeh Geranmayeh, Brenton Hordacre, Neda Jahanshad, Steven A Kautz, Bethany P Tavenner, Bradley J MacIntosh, Fabrizio Piras, Andrew D Robertson, Na Jin Seo, Surjo R Soekadar, Sophia I Thomopoulos, Daniela Vecchio, Timothy B Weng, Lars T Westlye, Carolee J Winstein, George F Wittenberg, Kristin A Wong, Paul M Thompson, Sook-Lei Liew, Amy F Kuceyeski

**Affiliations:** Department of Radiology, Weill Cornell Medicine, New York City, NY 10021, USA; Department of Neurology, Inselspital, University Hospital Bern, University of Bern, Bern 3012, Switzerland; Department of Radiology, Weill Cornell Medicine, New York City, NY 10021, USA; Department of Neurology, Carver College of Medicine, Iowa City, IA 52242, USA; Department of Neurology, Carver College of Medicine, Iowa City, IA 52242, USA; Department of Psychiatry, Carver College of Medicine, Iowa City, IA 52242, USA; Department of Pediatrics, Carver College of Medicine, Iowa City, IA 52242, USA; Department of Physical Therapy, Faculty of Medicine, The University of British Columbia, Vancouver, BC V6T 1Z4, Canada; Department of Sport, Exercise and Rehabilitation, Faculty of Health and Life Sciences, Northumbria University, Newcastle upon Tyne NE1 8ST, United Kingdom; Division of Physical Therapy, Department of Rehabilitation Medicine, Emory University School of Medicine, Atlanta, GA 30322, USA; Department of Physical Therapy, Faculty of Medicine, The University of British Columbia, Vancouver, BC V6T 1Z4, Canada; Djavad Mowafaghian Centre for Brain Health, University of British Columbia, Vancouver, BC V6T 1Z4, Canada; Department of Health Sciences, University of North Carolina at Chapel Hill, Chapel Hill, NC 27599, USA; Hospital das Clinicas HCFMUSP, Faculdade de Medicina, Universidade de Sao Paulo, Sao Paolo 05652-900, Brazil; Hospital Israelita Albert Einstein, São Paulo 05652-900, Brazil; Department Neurology, UCLA, California Rehabilitation Institute, Los Angeles, CA 90033, USA; Department of Neurology, Dell Medical School at The University of Texas Austin, Austin, TX 78712, USA; Clinical Language and Cognition Group, Department of Brain Sciences, Imperial College London, London W12 0HS, United Kingdom; Innovation, Implementation and Clinical Translation (IIMPACT) in Health, Allied Health and Human Performance, University of South Australia, Adelaide 5000, Australia; Imaging Genetics Center, Mark and Mary Stevens Neuroimaging and Informatics Institute, Keck School of Medicine, University of Southern California, Charleston, SC 29425, USA; Department of Health Sciences & Research, Medical University of South Carolina, Charleston, SC 29425, USA; Ralph H. Johnson VA Health Care System, Charleston, SC 29425, USA; Chan Division of Occupational Science and Occupational Therapy, University of Southern California, Los Angeles, CA 90033, USA; Sandra Black Centre for Brain Resilience and Recovery, Hurvitz Brain Sciences Program, Sunnybrook Research Institute, Toronto, ON M4N 3M5, Canada; Computational Radiology and Artificial Intelligence (CRAI), Department of Physics and Computational Radiology, Clinic for Radiology and Nuclear Medicine, Oslo University Hospital, Oslo 0372, Norway; Laboratory of Neuropsychiatry, Santa Lucia Foundation IRCCS, Rome 00179, Italy; Sandra Black Centre for Brain Resilience and Recovery, Hurvitz Brain Sciences Program, Sunnybrook Research Institute, Toronto, ON M4N 3M5, Canada; Schlegel-UW Research Institute for Aging, Waterloo, ON N2J 0E2, Canada; Department of Health Sciences & Research, Medical University of South Carolina, Charleston, SC 29425, USA; Ralph H. Johnson VA Health Care System, Charleston, SC 29425, USA; Department of Rehabilitation Sciences, Medical University of South Carolina, Charleston, SC 29425, USA; Department of Psychiatry and Neurosciences, Charité Campus Mitte (CCM), Charité—Universitätsmedizin Berlin, Berlin 10117, Germany; Imaging Genetics Center, Mark and Mary Stevens Neuroimaging and Informatics Institute, Keck School of Medicine, University of Southern California, Charleston, SC 29425, USA; Laboratory of Neuropsychiatry, Santa Lucia Foundation IRCCS, Rome 00179, Italy; Department of Neurology, Dell Medical School at The University of Texas Austin, Austin, TX 78712, USA; Department of Diagnostic Medicine, Dell Medical School, The University of Texas at Austin, Austin, TX 78712, USA; Department of Psychology, University of Oslo, Oslo 0372, Norway; NORMENT, Division of Mental Health and Addiction, Oslo University Hospital, Oslo 0372, Norway; Division of Biokinesiology and Physical Therapy, Herman Ostrow School of Dentistry, University of Southern California, Los Angeles, CA 90033, USA; Department of Neurology, Keck School of Medicine, University of Southern California, Los Angeles, CA 90033, USA; Department of Neurology, University of Pittsburgh, Pittsburgh, PA 15213, USA; Department of Bioengineering, University of Pittsburgh, Pittsburgh, PA 15213, USA; Department of Physical Medicine & Rehabilitation, University of Pittsburgh, Pittsburgh, PA 15213, USA; GRECC, HERL, Department of Veterans Affairs Pittsburgh Healthcare System, Pittsburgh, PA 15213, USA; Department of Physical Medicine & Rehabilitation, Dell Medical School, University of Texas at Austin, Austin, TX 78712, USA; Imaging Genetics Center, Mark and Mary Stevens Neuroimaging and Informatics Institute, Keck School of Medicine, University of Southern California, Charleston, SC 29425, USA; Stevens Neuroimaging and Informatics Institute, University of Southern California, Los Angeles, CA 90033, USA; Department of Radiology, Weill Cornell Medicine, New York City, NY 10021, USA

**Keywords:** machine learning, lesion-deficit associations, imaging biomarkers, stroke outcomes

## Abstract

Chronic motor impairments are a leading cause of disability after stroke. Previous studies have associated motor outcomes with the degree of damage to predefined structures in the motor system, such as the corticospinal tract. However, such theory-based approaches may not take full advantage of the information contained in clinical imaging data. The present study uses data-driven approaches to model chronic motor outcomes after stroke and compares the accuracy of these associations to previously-identified theory-based biomarkers. Using a cross-validation framework, regression models were trained using lesion masks and motor outcomes data from 789 stroke patients from the Enhancing NeuroImaging Genetics through Meta Analysis (ENIGMA) Stroke Recovery Working Group. Using the explained variance metric to measure the strength of the association between chronic motor outcomes and imaging biomarkers, we compared theory-based biomarkers, like lesion load to known motor tracts, to three data-driven biomarkers: lesion load of lesion-behaviour maps, lesion load of structural networks associated with lesion-behaviour maps, and measures of regional structural disconnection. In general, data-driven biomarkers had stronger associations with chronic motor outcomes accuracy than theory-based biomarkers. Data-driven models of regional structural disconnection performed the best of all models tested (*R*^2^ = 0.210, *P* < 0.001), performing significantly better than the theory-based biomarkers of lesion load of the corticospinal tract (*R*^2^ = 0.132, *P* < 0.001) and of multiple descending motor tracts (*R*^2^ = 0.180, *P* < 0.001). They also performed slightly, but significantly, better than other data-driven biomarkers including lesion load of lesion-behaviour maps (*R*^2^ = 0.200, *P* < 0.001) and lesion load of structural networks associated with lesion-behaviour maps (*R*^2^ = 0.167, *P* < 0.001). Ensemble models - combining basic demographic variables like age, sex, and time since stroke - improved the strength of associations for theory-based and data-driven biomarkers. Combining both theory-based and data-driven biomarkers with demographic variables improved predictions, and the best ensemble model achieved *R*^2^ = 0.241, *P* < 0.001. Overall, these results demonstrate that out-of-sample associations between chronic motor outcomes and data-driven imaging features, particularly when lesion data is represented in terms of structural disconnection, are stronger than associations between chronic motor outcomes and theory-based biomarkers. However, combining both theory-based and data-driven models provides the most robust associations.

## Teaser

Olafson *et al*. report that data-driven imaging biomarkers offer stronger associations with chronic motor outcomes after stroke than traditional theory-based biomarkers. The study demonstrates the superior accuracy of data-driven over theory-based approaches, with the best results achieved through ensemble models that integrate both types of biomarkers with demographic variables.

## Graphical Abstract

**Graphical abstract:**
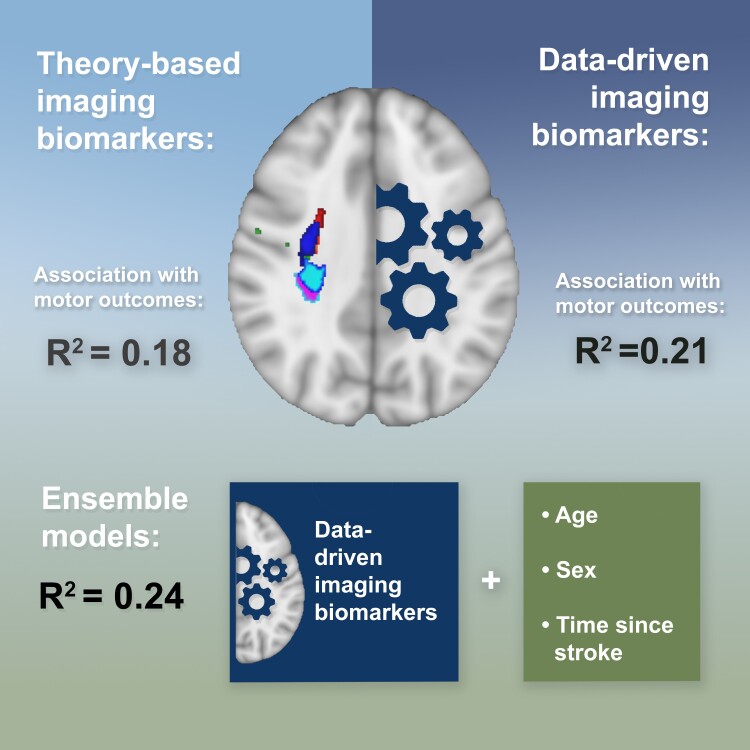

## Introduction

Motor impairments are the most common type of deficit after stroke, persisting in up to 50% of stroke survivors as lasting motor weakness.^1^ The nature and extent of deficits produced by a stroke are largely determined by its location, which can be determined with acute neuroimaging. Despite the prevalence of motor deficits after stroke, uncovering robust associations between neuroimaging measures and long-term motor deficits is still a challenge.^2,3^ Observational studies have demonstrated that motor impairments after stroke are associated with damage to critical structures in the motor system.^4-8^ Although these inferential studies help clarify the pathophysiology of motor deficits, few studies demonstrate that these patterns of association apply to new subjects, and in general, there is no consensus on how to optimally model lesion damage such that a machine-learning model can learn associations that are relevant for new subjects.

Historically, theory-based biomarkers selected *a priori* based on their involvement in motor function, have been used to model motor outcomes after stroke. The most well-studied theory-based biomarker is the corticospinal tract (CST) lesion load, or the proportion of voxels in the ipsilesional corticospinal tract originating from primary motor cortex (M1) that intersects with the lesion.^9-13^ M1-CST lesion load has been related to motor deficits in the acute and chronic phase of stroke,^3,14^ but M1-CST damage in itself may not capture enough variance in lesion data to explain motor deficits in patients with a wide range of lesion topographies.^11,15,16^ Incorporating measures of damage to higher-order motor structures into linear models (e.g. lesion load of all tracts in the sensorimotor tract template atlas, SMATT-LL) helps to explain more variance in post-stroke motor outcomes compared to models based on measures of damage to M1-CST alone.^11,15-20^ Although lesion load to these tracts has been significantly associated with motor deficits within individual samples, the out-of-sample association of theory-based biomarkers with motor scores has not been well-assessed.^14^

As an alternative to theory-based biomarkers, data-driven approaches assume that useful lesion-deficit associations can be discovered with sufficient data and proper representations of lesion damage.^21,22^ These approaches may produce more generalizable models than theory-based biomarkers: theory-based measures that are significantly related to motor outcomes in one sample may not necessarily associate with outcomes in a new sample.^23^ Whether such data-driven approaches have value in estimating stroke motor outcomes is unknown, and how to best represent lesion damage such that data-driven approaches can uncover generalizable lesion-deficit associations is unclear. One approach is to discover lesion-behaviour maps (LBMs) or voxels in which lesion damage is associated with motor deficits.^8,23^ Then, in a new sample, the extent of overlap between a patient's lesion and the LBM can be associated with motor outcomes. Similarly, the extent of lesion overlaps with structural lesion-network maps (sLNM), which reflect the white matter networks associated with peak LBM voxels, referred to previously as the sLNM lesion load,^8,23^ may be able to capture relationships between motor deficits and white matter tract damage. One limitation of using voxelwise representations of damage to develop lesion-behaviour maps is that non-overlapping lesions that impact the same white matter tract are treated separately, which may reduce the power of a model to identify robust lesion-deficit associations.^24^ Transforming voxelwise lesions into structural disconnection measures may better represent the neural correlates of post-stroke deficits and improve statistical power to detect critical features.^24^ This type of representation is, in effect, a non-linear dimensionality reduction of voxelwise lesion data that can collapse any damage along a white matter tract into a single feature. To this end, the Network Modification tool^25^ can be used to calculate lesions’ Change in Connectivity (ChaCo) scores, reflecting the amount of structural disconnection to/from each grey matter region in the brain, by identifying white matter tracts that pass through the lesion using structural connectomes from healthy subjects.^25^

We hypothesized that data-driven biomarkers would have stronger associations with chronic motor outcomes than theory-based biomarkers in new patients. Within data-driven biomarkers, we hypothesized that modelling lesion damage with whole-brain regional structural disconnection scores (ChaCo scores) would yield more accurate out-of-sample associations of chronic motor scores than modelling lesion damage with lesion-behaviour maps (LBM lesion load) and structural lesion-network maps (sLNM lesion load), but that sLNM-LL would perform better than LBM-LL due to the inclusion of relevant structural networks.

Accurate models of chronic motor outcomes require that patient information is combined across several data sources. In addition to lesion damage derived from imaging, demographic factors such as age, sex, and time since stroke influence an individual's chronic outcome. Models that employ combinations of imaging and demographic variables will likely be necessary for optimized associative models.^2^ Additionally, the strength of one imaging biomarker may be able to compensate for the weaknesses of others. Therefore, we hypothesized that association performance would be improved by incorporating demographic information and by combining point estimations from several different biomarkers using ensemble models.

The variability between stroke subjects owing to the significant heterogeneity within the disease poses a challenge for clinical trials to identify effective therapies, presenting a need to evaluate various biomarkers robustly associated with motor deficits.^26,27^

## Materials and methods

### Sample demographics

A subset of cross-sectional data from the Enhancing Neuroimaging Genomics through Meta Analysis (ENIGMA) Stroke Recovery Working Group database (available as of 10 September 2021) from subjects with acute/subacute and chronic stroke was used in the study (Table 1, Supplementary Table 1). In total, the dataset consisted of 327 acute/subacute stroke subjects and 462 chronic stroke subjects, totalling 789. Details of the ENIGMA Stroke Recovery procedures and methods are available in Liew *et al*.^28^ The data originated from 22 research studies carried out at different sites. Informed consent was obtained from all subjects, and data were collected in compliance with each institution's local ethical review boards and in accordance with the Declaration of Helsinki.

**Table 1:** Demographic information of subjects with chronic stroke (*N* = 462) in the ENIGMA dataset, by site

Site ID	Total N. *N* (F/M)	Median age in years (IQR)	Median motor score (IQR)	Median time since stroke	Median lesion vol. in *cm*^3^ (IQR)
r001	39 (10/29)	61.0 (17.0)	0.65 (0.23)	23.5 (40.0)	6.27 (18.06)
r002	12 (6/6)	69.5 (11.5)	0.50 (0.41)	73.2 (51.9)	28.24 (31.71)
r003	15 (6/9)	61.0 (16.5)	0.24 (0.20)	48.8 (67.6)	20.28 (76.88)
r004	19 (7/12)	44.0 (14.5)	0.17 (0.16)	50.4 (81.9)	36.85 (44.29)
r005	27 (12/15)	66.0 (16.5)	0.79 (0.45)	31.4 (27.8)	1.61 (40.27)
r009	60 (17/43)	71.0 (7.2)	0.96 (0.12)	27.4 (9.3)	1.43 (4.65)
r025	16 (3/13)	64.5 (13.2)	0.98 (0.58)	14.2 (10.2)	5.92 (14.26)
r027	28 (8/20)	57.0 (10.2)	0.30 (0.16)	19.3 (24.7)	12.30 (62.13)
r028	21 (6/15)	63.0 (9.0)	0.82 (0.24)	26.5 (37.5)	5.25 (41.28)
r031	1 (0/1)	52.0 (0.0)	0.68 (0.00)	6.1 (0.0)	1.54 (0.00)
r034	15 (6/9)	58.4 (11.1)	0.82 (0.20)	61.3 (68.3)	6.68 (34.99)
r035	15 (6/9)	64.0 (18.0)	0.64 (0.52)	33.5 (22.9)	3.89 (31.56)
r038	18 (7/11)	67.0 (10.0)	1.00 (0.12)	15.1 (10.1)	1.98 (1.63)
r040	14 (7/7)	63.5 (9.8)	0.68 (0.47)	14.1 (17.5)	8.65 (82.65)
r042	22 (11/11)	48.5 (15.5)	0.64 (0.19)	29.6 (36.4)	14.16 (49.53)
r044	4 (0/4)	68.0 (9.2)	0.52 (0.25)	43.7 (52.9)	23.65 (67.00)
r045	4 (1/3)	62.0 (5.2)	0.49 (0.24)	96.1 (59.0)	7.97 (6.66)
r046	11 (3/8)	62.0 (10.5)	0.50 (0.29)	86.3 (83.4)	4.62 (19.82)
r047	44 (14/30)	65.5 (12.0)	0.65 (0.44)	38.1 (53.7)	12.72 (41.33)
r048	43 (16/27)	68.0 (12.5)	0.79 (0.44)	46.2 (49.8)	7.93 (43.45)
r052	32 (12/20)	63.0 (13.5)	0.41 (0.09)	39.1 (42.2)	6.98 (51.55)
r053	2 (1/1)	65.0 (3.0)	0.63 (0.25)	6.2 (0.1)	117.9 (17.39)

Some sites have both acute and chronic subjects that are listed separately. Total sample size (N), number of females (F) and males (M), and information about age (years), normalized motor scores, time since stroke at the time of assessment (months), and lesion volume (*cm*^3^). IQR, interquartile range.

ENIGMA Stroke Recovery subjects with the following data were included: (1) high-resolution (1-mm isotropic) T1-weighted brain MRI (T1w) acquired with a 3T MRI scanner; (2) information about time since stroke at the time of imaging, as well as (3) age, (4), sex, and (5) a measure of sensorimotor function from one of the following assessments: (i) Fugl–Meyer Assessment of Upper Extremities (FMA-UE), a performance-based measure of paretic upper extremity impairment,^29^ (ii) the Barthel index, which measures the extent to which a person can function independently and has mobility in their activities of daily living,^30^ or (iii) the National Institutes of Health Stroke Score (NIHSS), a broad measure of stroke severity that includes assessment of non-motor and motor functions.^31^ For most subjects with chronic stroke, motor deficits are normalized FMA-UE scores, whereas normalized FMA-UE scores were available for fewer of the subjects with acute/subacute stroke (Supplementary Table 2); for simplicity, we refer to all outcomes as ‘motor’ scores, though the main models were replicated with a single motor assessment (Fugl–Meyer UE scores) as well. Motor scores were normalized to the range [0, 1] by dividing the raw score by the maximum possible score for that assessment. Behavioural data were collected within approximately 72 hours of the MRI. Subjects were considered in the chronic phase of stroke if their time since stroke at the time of assessment was greater than or equal to 180 days and were considered to be in an acute/subacute phase if they were assessed within 180 days after the stroke. See Supplementary Fig. 1 for lesion distribution in Montreal Neurological Institute (MNI) space.

Lesions were manually segmented via a standardized protocol,^32^ and segmentations were reviewed by two additional team members.^32,33^ Image processing was performed as follows (as described previously^33^): intensity normalization with the MINC-toolkit and registration of the T1w image and lesion segmentation map to a standardized template (*MNI152NLin2009aSym,* 2 mm), followed by defacing.^33^ Registration quality was confirmed by visual inspection to ensure correct alignment to the template.^33^

### General overview

We built several models to associate chronic motor scores with imaging data and minimal demographic information. Each model used lesion-derived information as input to estimate normalized motor scores in subjects with chronic stroke. We compared several different biomarkers that reflect different aspects of lesion damage. These biomarkers include:

**Theory-based biomarkers:**

- M1-CST-LL (1 feature)
- SMATT-LL (6 features for ipsilesional tracts, 12 features for bilateral tracts)

**Data-driven biomarkers:**

- LBM-LL (1 feature)
- sLNM-LL (five features; five components derived from principal components analysis of structural connectivity seeded from lesion-behaviour map)
- ChaCo scores (86 or 268 features depending on the atlas, fewer when using feature selection).

Nested cross-validation was performed during model training and performance was assessed on unseen test data. For ChaCo score models, we assessed whether feature selection improved performance. Additionally, we evaluated whether including acute subjects in the training set, but not in the test set, improved association strength with chronic deficits. We also evaluated whether adding basic demographic information (age, sex, and time since stroke) via ensemble models improved performance. We also assessed whether using ensemble models to combine point estimations from multiple different lesion damage metrics improved performance. Finally, we evaluated the performance of each model on completely unseen data by removing sites from the training set and using them as test sets.

### Machine-learning framework

**Figure 1:**
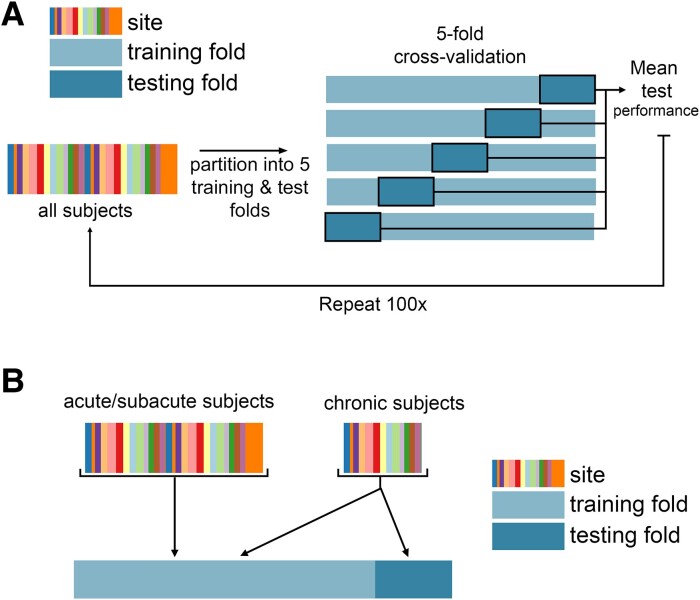
**Cross-validation framework for model evaluation. A.** Overview of five-fold cross-validation. Subject data are partitioned into five non-overlapping training and test folds, such that no training subjects are in the test set, and no subject is in the test fold more than once. **B.** Use of acute/subacute subjects in training folds but not test folds. When using all training data, chronic subjects were included in the test folds and training folds, whereas acute/subacute stroke subjects were only included in training folds.

Regression models were trained and evaluated using repeated 5-fold nested cross-validation (Fig. 1). Model implementation differed for each lesion biomarker based on the dimensionality of the data; see below for implementation details for each biomarker. For biomarkers with more than one feature, ridge regression models were fit with a single hyperparameter indicating the degree of regularization. In the outer loop, the data were split into 5 training and test partitions. First, models were trained using all data (including acute, subacute, and chronic timepoints), and tested only on chronic subjects, yielding 696 subjects in the training set and 92 subjects in the test set. Using only chronic data to train the models, there were approximately 370 subjects in the training set and 92 subjects in the test set. Out-of-sample performance was calculated as the average performance across five outer test folds. We obtained a distribution of out-of-sample performance by splitting the data into 5 train/test folds 100 times, shuffling the indices of the splits each time.

### Replication with Fugl–Meyer assessments in unseen sites

Although subjects who were included in the training set were never included in the test set for each fold, it is possible that subjects in the training set share some features with the test set, simply by coming from the same site. Therefore, in order to measure the models’ generalizability to out-of-distribution data, we performed additional analyses by leaving entire sites out of the training set and using only those sites in the outer test fold. For these additional analyses, we further limited the test set to only include subjects if their outcome measures were Fugl–Meyer Assessments, as that score is most reflective of motor function which our models are focused on estimating. Three folds were created with approximately equal numbers of subjects (*N* = 121, 127, and 130), where only subjects who were considered to be in the chronic stage of stroke were included (Supplementary Table 4).

### Statistical analysis

#### Model performance

Model performance was assessed by comparing true normalized motor scores with estimated scores. Performance was calculated with both Pearson's correlation coefficient and explained variance, or *R*^2^, which captures the per cent of variation in motor scores explained by variation in the model inputs:


$$R^{2}=1-\mathrm{var}(y-\hat{y})/\mathrm{var}(y)$$

where *y* is a vector of true motor outcomes, and $\hat{y}$ is a vector of estimated motor outcomes. These two performance metrics were used to compare results with prior literature, but differences between models were assessed using *R*^2^, as it is a more robust metric to assess model quality.

Differences in performance between models were assessed using two-sided Wilcoxon signed-rank tests and *P*-values were corrected for multiple comparisons using Bonferroni correction (*P* < 0.05).

For each model, we generated a null distribution for assessing model significance by permuting the estimated variable (motor score) 100 times. Then, as for each normal model, we ran 100 5-fold train/test cross-validation splits for each permutation. This yielded a distribution of 100 out-of-sample mean performance measures for each permutation. The median across these 100 measures was then calculated for each permutation. In total, 100 null median performance measures were calculated. The *P*-value for the model's significance is the proportion of null models that had a median *R*^2^ greater than or equal to the median performance of the true model.

#### Ensemble models

The idea of ensemble learning is to build a single model by combining the strengths of a collection of simpler base models; we used ensemble models that average point estimations from different biomarkers.^34^ We tested whether models including demographic information (age, sex, and days post stroke), ensembled with lesion models, performed better than models with lesion data or demographic data alone. We also assessed whether models including both lesion load and ChaCo scores would perform better than models with lesion load or ChaCo scores alone. Ensemble models were generated by training ChaCo models and lesion load models separately, on the same subjects and with the same training/test/validation splits, and averaging the final estimated scores for each test subject. Standard linear regression was used to model the relationship between demographic information and motor impairment.

#### Analysing feature weights

Feature weights in high-dimensional models can be unstable and therefore only provide limited interpretability.^35^ To assess the robustness of feature weights (i.e. beta coefficients), the Pearson correlation in regional feature weights across all training folds was calculated. For this specific feature stability analysis (but not for model evaluation), acute/subacute subjects were split for each training fold such that different folds did not contain the same set of acute/subacute subjects (all acute/subacute subjects were included in all training folds in the model evaluation phase to maximize the amount of data available for training). For the 86-region Chaco models, the average of the 86-region *β* vector, representing the model weights for each region, across five folds was calculated for each of the 100 train/test splits. The median across these 100 splits was visualized on a glass brain. For the 268-region ChaCo models with feature selection, the most consistently selected regions (selected in at least 475/500 folds or 99% of outer folds) were identified. The average of the 268-region *β* vector across the five folds was calculated for each of 100 train/test splits. The median average *β* weight for consistently selected regions across these 100 splits was visualized on a glass brain.

### Description of models and their inputs

#### Primary motor cortex CST lesion load (M1-CST-LL) models

**Figure 2:**
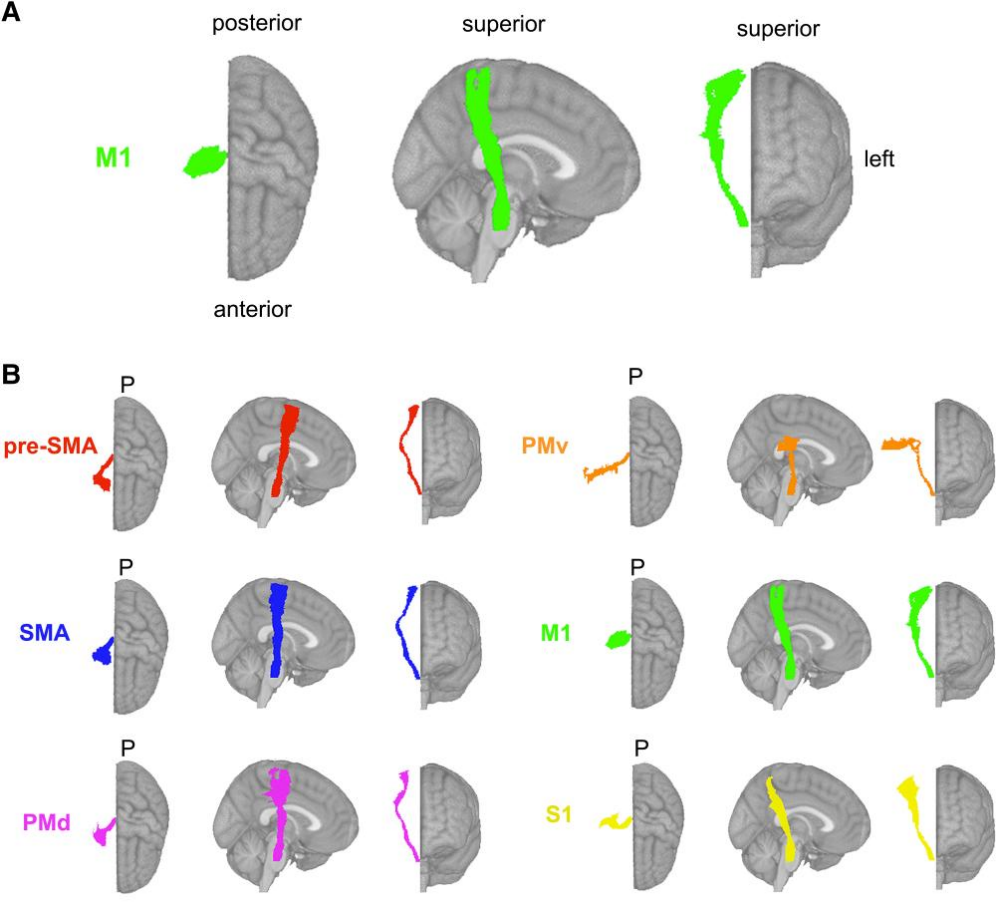
**Theory-based biomarkers. A.** The M1-CST, displaying only the right hemisphere tracts relative to an MNI (Montreal Neurological Institute) template. **B.** Tracts from the sensorimotor tract template atlas (SMATT), displaying only right hemisphere tracts relative to an MNI template, including pre-supplementary motor area (pre-SMA), supplementary motor area (SMA), dorsal premotor cortex (PMd), ventral premotor cortex (PMv), primary motor cortex (M1), and primary sensory cortex (S1). Pre-SMA is the most anterior tract, and S1 is the most posterior tract.

The lesion load of the corticospinal tract originating from the primary motor cortex (M1-CST-LL) was calculated (Fig. 2A). Here, as in previous work, M1-CST-LL was calculated as the proportion of lesioned voxels intersecting with a binarized ipsilesional M1-CST template.^9^ Specifically, lesion load was calculated in 1-mm MNIv6 space as follows:


$$\text{Lesion load}=\frac{Number\;of\;lesioned\;voxels\;intersecting\;withtract}{Number\;of\;voxels\;in\;tract}$$

Left and right hemisphere M1-CST segmentations in MNI space were obtained from the high-resolution sensorimotor area tract template (SMATT).^36^ Few subjects had non-zero M1-CST-LL values (Supplementary Fig. 2A). Linear regression was used to model the relationship between ipsilesional M1-CST-LL and chronic motor scores. The weights from the best-performing model in the inner loop were used to estimate motor scores for new subjects in the test folds.

#### Sensorimotor tract lesion load (SMATT-LL) models

Sensorimotor tract segmentations were obtained from the sensorimotor area tract template (SMATT),^36^ which contains 12 tracts derived from probabilistic tractography seeded in the left and right primary motor cortex (M1), dorsal and ventral premotor cortex (PMd and PMv, respectively), supplementary motor area (SMA), pre-supplementary motor area (pre-SMA), and primary somatosensory cortex (S1) performed in healthy controls (Fig. 2B).

Lesion load was calculated as above for all 12 bilateral tracts (L/R SMATT-LL) and for six ipsilesional tracts (ipsilesional SMATT-LL). L/R SMATT-LL was calculated in order to assess whether preserving hemispheric information improved associations.^37^ For subjects with brainstem, cerebellar, and/or bilateral cerebral strokes, ipsilesional lesion load was calculated as the average lesion load of the left and right hemisphere tracts (Supplementary Fig. 2B and C).

Ridge regression models were used to estimate chronic motor deficits from ipsilesional SMATT- LL (6 features) and from L/R-SMATT-LL (12 features). Ridge regression was used to account for multicollinearity of lesion load values between tracts (Supplementary Figs. 3 and 4). Lesion load values were normalized (after train/test split) by subtracting the mean across subjects and dividing by the l2-norm prior to model fitting. In the inner loop, the degree of model regularization (*λ*) was determined via grid search over 30 values ranging from 10*^−^*^2^ to 102. The training data were fit with the selected *λ*, and this model was used to estimate motor scores for held-out subjects in the test folds.

#### Lesion-behaviour map lesion load (LBM-LL) models

**Figure 3:**
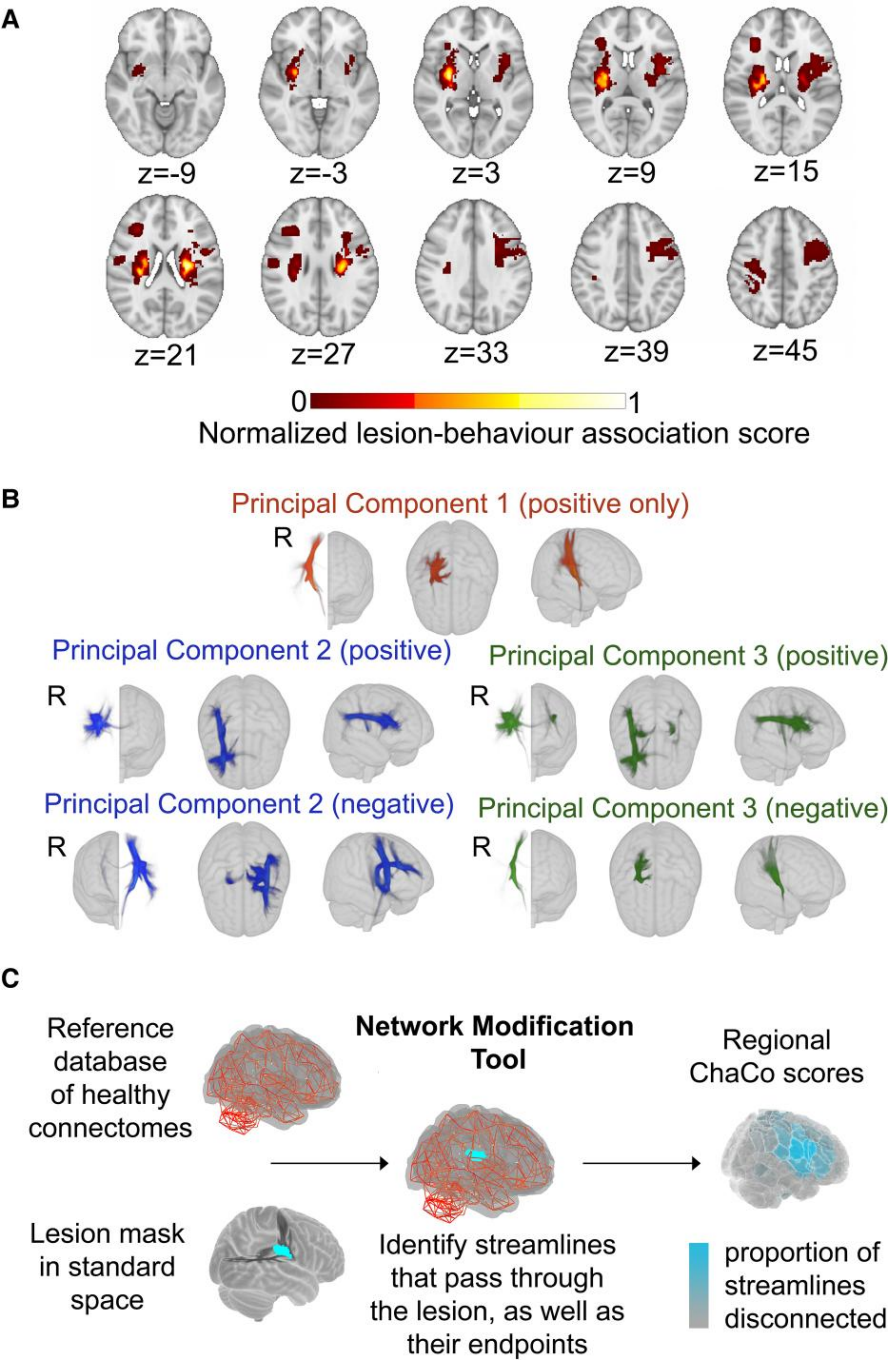
**Data-driven biomarkers. A**. Lesion-behaviour map (LBM) representing the association between voxelwise damage and Fugl–Meyer scores, derived from multivariate lesion-behaviour mapping with Fugl–Meyer scores. **B.** Structural lesion-network maps (sLNMs), derived from seed-based tractography run on peak regions identified from LBM (**A**) and then performing principal components analysis to identify 3 components, split into positive and negative weights. **C.** Change in Connectivity (ChaCo) scores derived from the Network Modification (NeMo) tool. Binary lesion masks in MNI space representing the presence of a stroke lesion (turquoise) in a given voxel are provided by the user. Each lesion mask is embedded into 420 unrelated healthy structural connectomes (separately for each healthy subject) and the regional ChaCo scores are calculated and averaged across healthy subjects (parcellation shown here is the Shen 268-region atlas).

A lesion-behaviour map (Fig. 3A) was obtained as described by Bowren *et al*. (2022). Specifically, Bowren *et al*. used sparse canonical correlation analysis to produce maps of voxels in which damage was associated with Fugl–Meyer scores.^38^ Lesion load to this lesion-behaviour map (LBM-LL) was calculated as the sum of voxels in the LBM that intersect with the lesion. Standard linear regression models were used to estimate chronic motor deficits from LBM-LL.

#### Structural lesion-network mapping lesion load (sLNM-LL) models

Structural lesion-network maps (Fig. 3B) were obtained from Bowren *et al*. (2022). Specifically, peak white matter (WM) voxels from lesion-behaviour maps (described above) were identified. Then, tractography was seeded from these peak WM voxels to identify associated structural networks, called structural lesion-network maps (sLNMs). Principal components analysis of sLNMs was performed, which produced three principal components that correspond to five sLNM maps (PC1, and positive/negative weights of PC2 and PC3). Lesion load on each sLNM map was calculated for each subject as the sum of the voxel intensities from the principal component map that intersected the lesion mask (Supplementary Fig. 5). Ridge regression models were used to estimate chronic motor deficits from sLNM lesion loads (five features).

#### Regional change in connectivity (ChaCo) models

Lesion masks in 1-mm^3^ MNI v6 space were processed with the Network Modification Tool (NeMo Tool) v2 pipeline,^25^ available at https://kuceyeski-wcm-web.s3.us-east-1.amazonaws.com/upload.html; see https://github.com/kjamison/nemo for documentation. Given a lesion mask, the NeMo tool produces outputs that reflect the impact of the lesion on white matter tracts using healthy structural connectomes as a reference. The NeMo tool embeds a lesion mask into healthy structural connectomes, identifies all white matter streamlines that intersect with the lesion, and determines the brain regions at the endpoints of those streamlines (Fig. 3C). Regional change in connectivity (ChaCo) scores, or the ratio of the number of disrupted streamlines divided by the total number of streamlines terminating in each region, was calculated for all grey matter regions (see Supplementary Fig. 6A and B for distribution of mean and standard deviation of ChaCo scores). The NeMo tool uses structural connectivity from 420 unrelated subjects from the Human Connectome Project (HCP) Young Adult database. Regional ChaCo scores from two different atlases were compared: the 86-region Desikan-Killiany Atlas (68 cortical regions + 18 subcortical regions, excluding brainstem) from FreeSurfer (“fs86” for short), which contains coarse anatomically parcellated regions,^39,40^ and the 268-region Shen atlas (“shen268” for short), which contains more fine-grained functionally parcellated cortical and subcortical regions.^41^

First, the performance of ridge regression models was assessed, as described above, with regional ChaCo scores as inputs (86 features for the fs86 atlas, 268 features for the shen268 atlas). Then, a filter-based feature selection step was added to the ridge regression models to obtain a subset of features that had the strongest association with the outcomes.^42^ Features were ranked by their association with the outcome variable (*P*-value from univariate regression) and only the *κ* most associated variables were included in the model. In the inner hyperparameter selection loop, both the amount of regularization on regression coefficients (*λ*) and the number of features to retain in the model (*κ*) were selected via grid search. The *λ* value was chosen by searching over 30 values ranging 10*^−^*^2^ to 10^2^, and the *κ* value was chosen by searching 30 values ranging from 5 to the maximum number of features possible (for fs86: 86, for shen268: 268).

### Code availability

The scikit-learn package was used to implement machine-learning models (http://scikit-learn.org). All analysis scripts that generated the results of the present study are available as open source (https://github.com/emilyolafson/lesion_predictions), and the LBM and sLNM maps are also available on the repository.

## Results

### Relative performance of models

**Figure 4:**
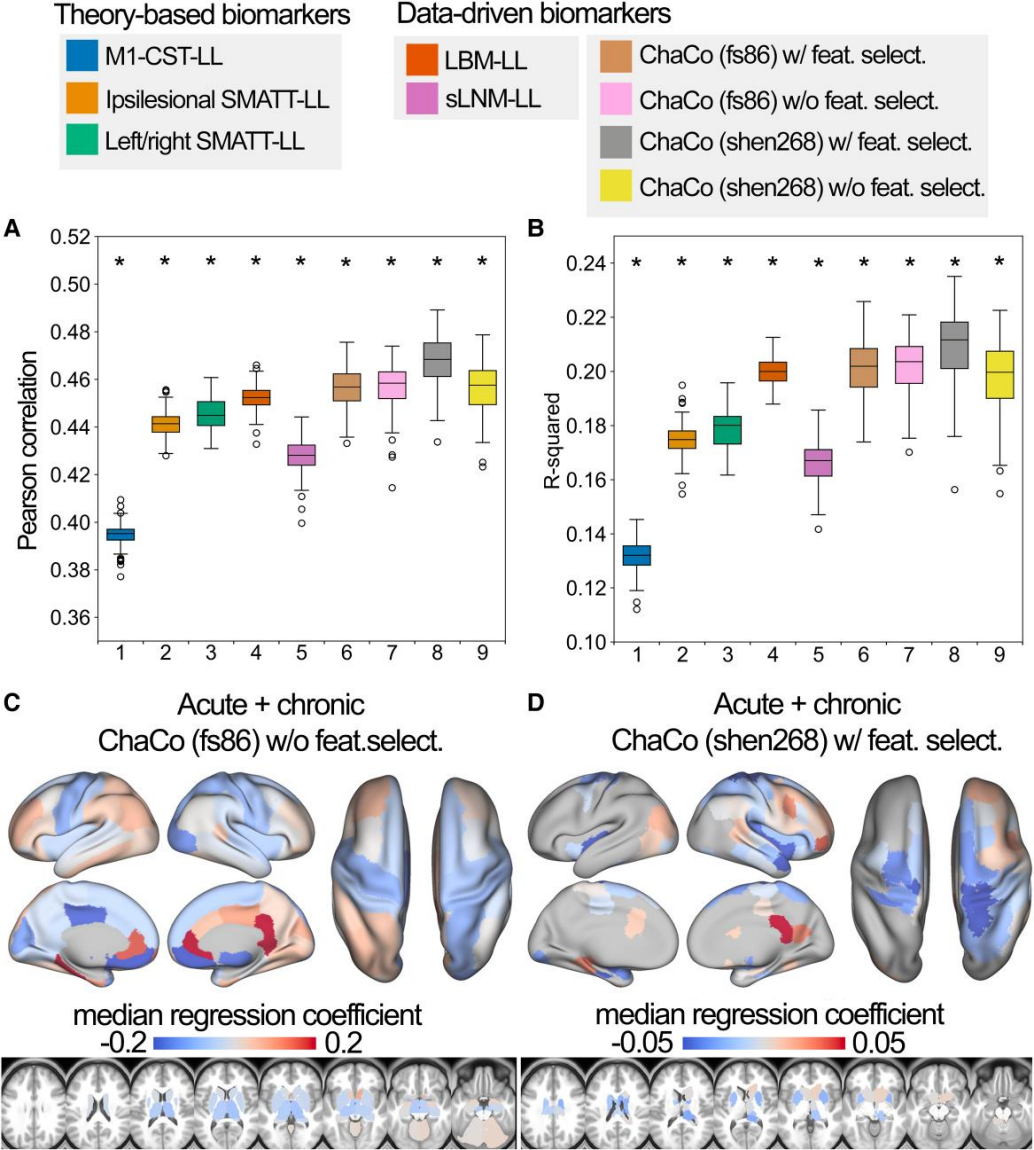
**Summary of model performance metrics across all models tested and feature weights (regression coefficients *β*) for the two best-performing models. A.** and **B.** Distribution of model performance (mean Pearson correlation/*R*^2^ across five outer folds for 100 permutations of the data, *N* = 92 for each fold). Asterisks (*) indicate that model performance is significantly above chance (*, P < 0.001), as assessed via permutation testing, where the *P*-value for the model's significance is the proportion of null models that had median *R*^2^ greater than or equal to the median performance of the true model. The boxes extend from the lower to upper quartile values of the data, with a line at the median. Whiskers represent the range of the data from (Q1-1.5*IQR, Q3 + 1.5*IQR). **C.** and **D.** Mean feature weights for the top two best-performing models (ChaCo (fs86) without feature selection, ChaCo (shen268) with feature selection, respectively). For the fs86-ChaCo model (left), we display the mean regression coefficients *β* across 100 permutations. For ChaCo (shen268) (right), we display the median regression coefficients of regions that were selected in at least 95% of outer folds (i.e. for regions that were included in the model in at least 475/500 outer folds, mean *β* coefficients were calculated across five outer folds, and the median value across 100 permutations is plotted).

**Figure 5:**
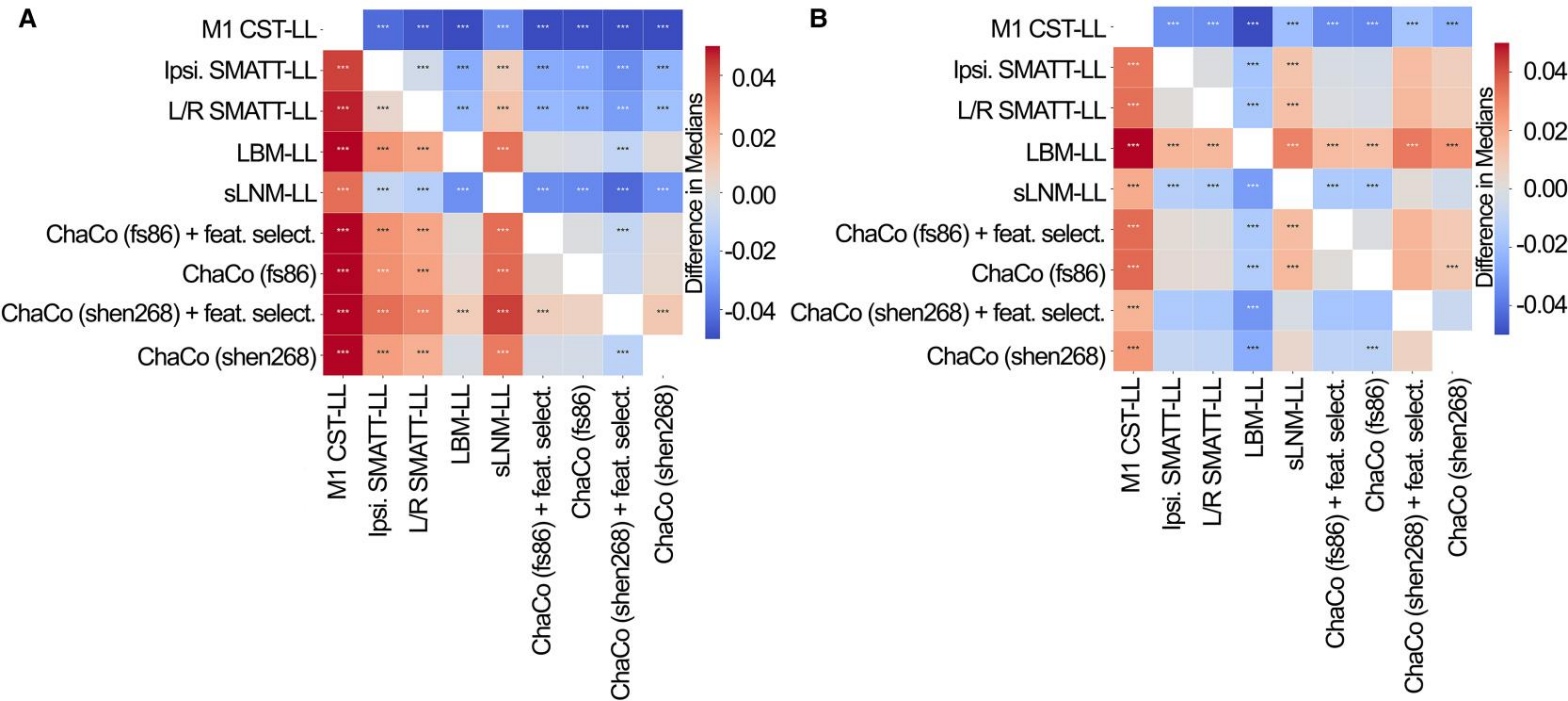
**Statistical comparison of model performance for estimating motor scores using Mann-Whitney signed-rank tests. Colours shown indicate the differences in median explained variance scores for each model. A.** Models trained using all (acute and chronic) training data. **B**. Models trained only using chronic data. *** denotes corrected *P* < 0.001 after Bonferroni correction. A positive difference indicates that the model on the *y*-axis (vertical) has a greater explained variance than the model on the *x*-axis (horizontal).

The out-of-sample performances of the models using all training data can be found in Fig. 4A and B. All models performed significantly better than chance (*P* < 0.001). With the exception of sLNM-LL models, all data-driven models (i.e. LBM-LL and ChaCo models) outperformed all theory-based models when using all training data (Fig. 5A). When using only chronic data for training, only LBM-LL models outperformed all theory-based models (Fig. 5B, Supplementary Fig. 7A and B).

Within the theory-based biomarkers, M1-CST-LL models performed worse than ipsilesional SMATT-LL models (difference in *R*^2^ = −0.043, *P* < 0.001, 95% CI [*−*0.044*, −*0.041]) and worse than left/right SMATT-LL models (difference in *R*^2^ = −0.047, *P* < 0.001, 95% CI [*−*0.049*, −*0.045]).

Within the data-driven biomarkers, models using ChaCo scores parcellated with the Shen 268-region atlas and with correlation-based feature selection outperformed LBM-LL models (difference in *R*^2^ = −0.010, *P* < 0.001, 95% CI [*−*0.013*, −*0.007]). However, ChaCo models performed comparably to LBM-LL models when using only chronic training data (Supplementary Fig. 7A and B). Using all training data, all ChaCo models outperformed sLNM-LL models. When using only chronic training data, the differences between sLNM-LL models and ChaCo scores parcellated with the 268-region atlas were non-significant (Supplementary Fig. 7A and B). sLNM-LL models performed worse than LBM-LL models using all training data (difference in *R*^2^ = −0.034, *P* < 0.001, 95% CI [*−*0.035*, −*0.032]) and chronic training data (difference in *R*^2^ = −0.029, *P* < 0.001, 95% CI [*−*0.031*, −*0.028]).

**Figure 6:**
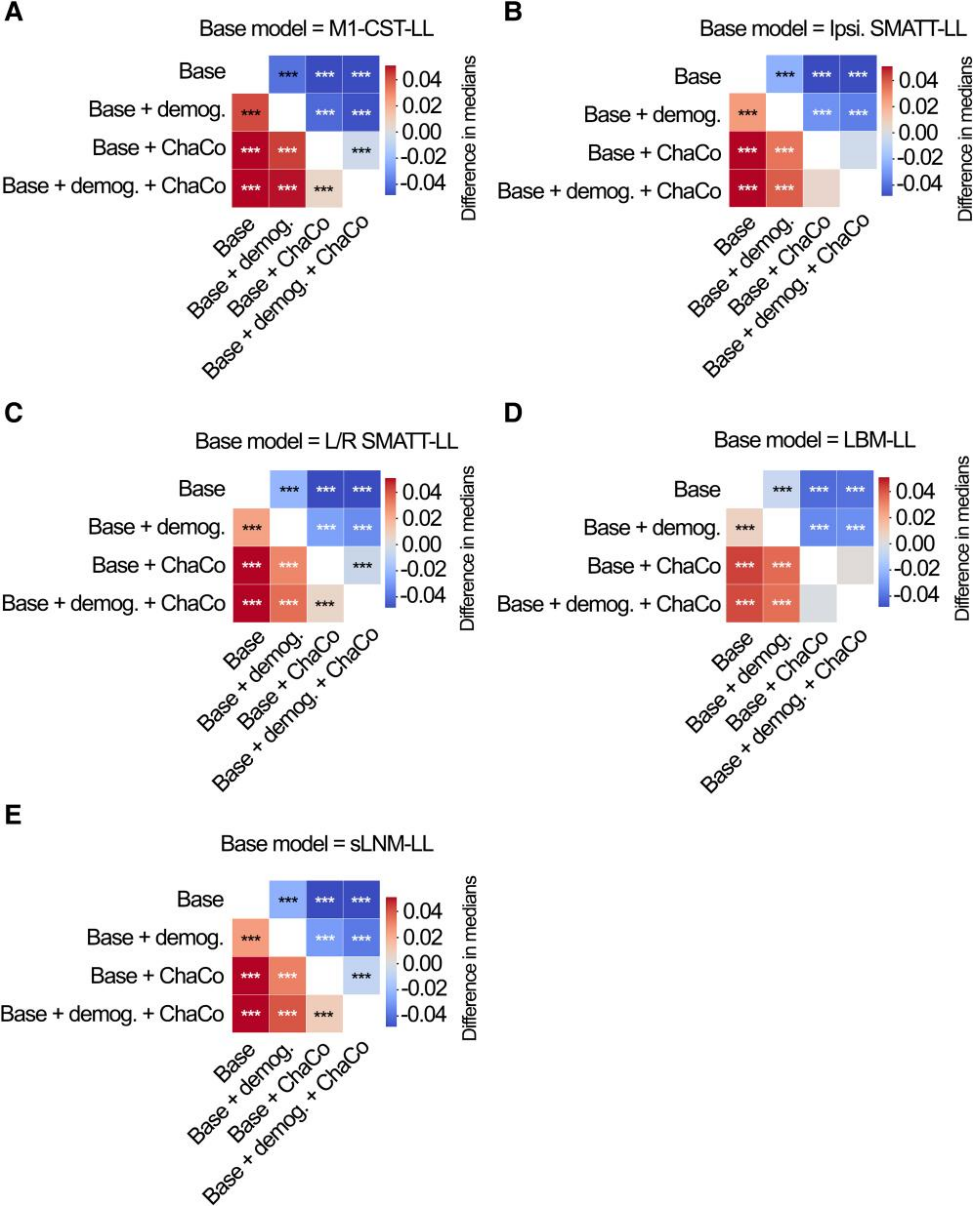
**Statistical comparison of model performance for ensemble models.** Demog. = demographic information (age, sex, days since stroke). ChaCo = model using 268-region ChaCo scores w/ feature selection. Significance of differences in explained variance was evaluated using Mann-Whitney signed-rank tests; ***denotes corrected *P* < 0.001 after Bonferroni correction. A positive difference value indicates that the model on the y-axis (vertical) has a greater explained variance than the model on the x-axis (horizontal). Panels A-E display differences in model performance relative to (**A**) M1-CST-LL, (**B**) Ipsilesional SMATT-LL, (**C**) left/right hemisphere SMATT-LL, (**D**) LBM-LL, and (**E**) sLNM-LL.

For all models tested, ensemble models combining point estimates from demographic data had stronger associations with motor outcomes than base models (Fig. 6A-E, Supplementary Table 3). Similarly, ensemble models merging estimates with the best-performing ChaCo models performed better than base lesion load models. With the exception of LBM-LL models, ensemble models combining information from demographic data as well as ChaCo scores performed best (Fig. 6A-E, Supplementary Table 3). The best overall ensemble model included LBM-LL and 268-region ChaCo scores with feature selection.

### Estimating Fugl–Meyer scores in held-out sites

To test whether models were generalizable to entirely new sites and to compare models in their ability to predict a measure that measures purely motor impairment—the Fugl–Meyer assessment—sites were held out to form three unique test sets. We observed similar performances in these test folds in comparison to the main analyses (Table 2). For two out of three test folds (Folds 1 and 2), all models captured some variance in the data, with data-driven models performing best: LBM-LL (*R*^2^ = 0.255) and 268-region ChaCo scores with feature selection (*R*^2^ = 0.240). For the remaining test fold, although all models performed poorly (*R*^2^ ranging from −0.175 to 0.03, indicating poor model fit), the model using LBM-LL scores performed best (*R*^2^ = 0.03).

**Table 2:** Performance of models estimating Fugl–Meyer scores

Fold	Model	*R* ^2^ score	Correlation
1	M1-CST-LL	0.155	0.419
1	Ipsilesional SMATT-LL	0.122	0.419
1	Left/right SMATT-LL	0.153	0.435
1	LBM-LL	**0.255**	0.506
1	FS86 (with feat. select.)	0.134	0.393
1	Shen268 (with feat. select.)	0.088	0.35
2	M1-CST-LL	0.035	0.378
2	Ipsilesional SMATT-LL	0.129	0.456
2	Left/right SMATT-LL	0.075	0.417
2	LBM-LL	0.165	0.433
2	FS86 (with feat. select.)	0.209	0.478
2	Shen268 (with feat. select.)	**0.240**	0.501
3	M1-CST-LL	−0.175	0.06
3	Ipsilesional SMATT-LL	−0.013	0.207
3	Left/right SMATT-LL	0.002	0.222
3	LBM-LL	**0.003**	0.239
3	FS86 (with feat. select.)	−0.114	0.234
3	Shen268 (with feat. select.)	−0.096	0.218

Displaying *R*^2^ and correlation of hold-out performances on the three test folds, each containing entire sites not used in training. Bold entries indicate the best performance across the fold.

### Featured selected by ChaCo models

Model weights for the best-performing ChaCo models are shown in Fig. 4C and D, reflecting the median regression weight for each region across 100 train/test splits. There were several spatial similarities in the pattern of regression weights for the 86-region ChaCo model and 268-region ChaCo model with feature selection. For both atlases, negative model weights (indicating that more disconnection is associated with worse motor outcomes, holding all other factors constant) are assigned to the left and right motor areas, as well as subcortical structures like the putamen and thalamus, whereas positive weights are assigned to frontal, parietal, and cingulate areas. In the 268-region ChaCo models, more regions in the right hemisphere are consistently included in the model than in the left hemisphere.

**Figure 7:**
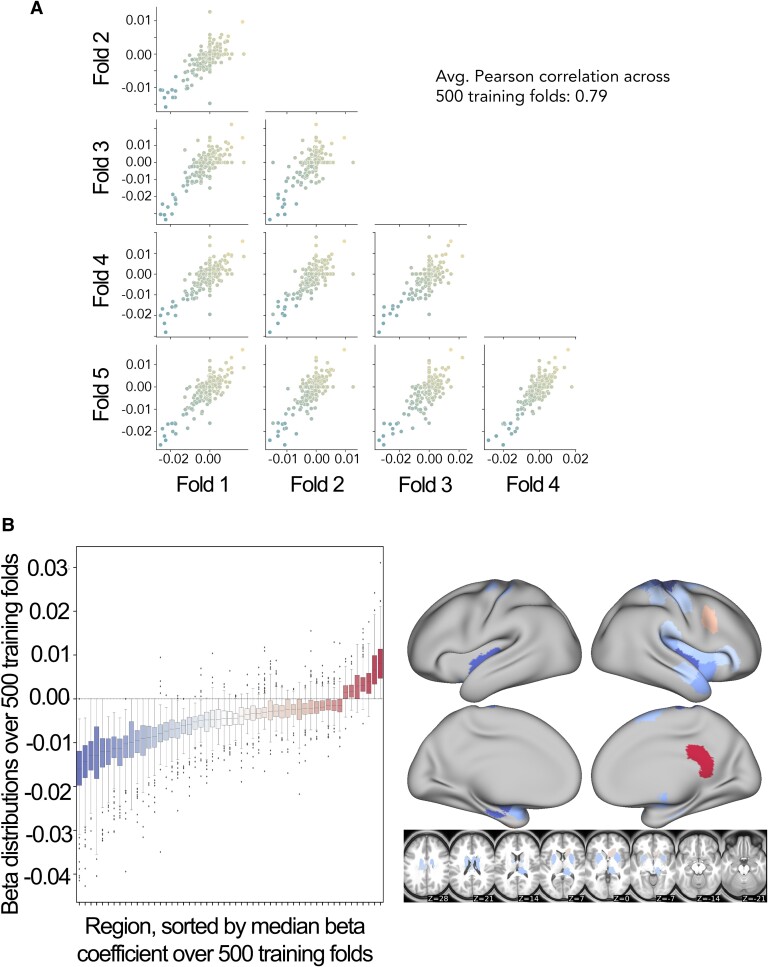
**Analysis of feature stability for 268-region ChaCo models (with feature selection) and investigation of paradoxical feature weights. A**. Scatter plots displaying similarity between beta coefficients across five training folds for one permutation. Each point corresponds to one region, and points are coloured by the mean beta coefficient for that region across 500 training folds (i.e. coloured based on *y*-axis value). The average Pearson correlation coefficient across 500 folds is reported. **B**. Boxplots show the distribution of beta coefficients of consistently weighted regions (defined as having median beta coefficients that are zero or of an opposite sign <5% of the time). In total, 30 regions with consistent negative weights and five regions with consistent positive weights remained. Median weights for consistently weighted regions are plotted on a brain. The boxes extend from the lower to upper quartile values of the data, with a line at the median. Whiskers represent the range of the data from (Q1–1.5*IQR, Q3 + 1.5*IQR).

The average correlation in feature weights between training folds was stable for the 268-region ChaCo score models, with an average *r* = 0.79 (Fig. 7). Furthermore, many regions with high-magnitude median feature weights had consistent weights across training folds. Finally, we observed evidence that 268-region ChaCo models were able to distinguish two regions’ relationships to motor scores, despite those regions being frequently damaged together (Supplementary Fig. 8).

## Discussion

In this study, we compared the performance of several structural imaging biomarkers in their association with post-stroke motor scores. We found that, in general, data-driven models performed better than theory-based models in their ability to associate with motor deficits in out-of-sample data, and this was replicated with a subset of the original data when estimating only FMA-UE scores. Among the data-driven models, we found that the best performance was obtained by modelling lesion damage using regional ChaCo scores. Contrary to our hypothesis, models using lesion-behaviour maps performed significantly better than structural lesion-network maps. Finally, we saw that combining estimates from demographic information and combining multiple biomarkers improved the association strength with post-stroke motor ability over baseline models.

### Data-driven biomarkers outperform theory-based biomarkers

Using all training data, the best-performing data-driven models used regional structural disconnection scores. These models, in addition to another data-driven biomarker, the extent of lesion damage to lesion-behaviour maps, outperformed all theory-based biomarkers. Additionally, data-driven models performed best when estimating FMA-UE scores in entire held-out sites, though all imaging biomarkers (theory-based and data-driven) did not associate as strongly in one of three hold-out sets, possibly due to a strong distribution shift for this test set.

Data-driven biomarkers may have outperformed theory-based biomarkers for two reasons. First, there may be regions outside of the primary motor system where lesions have an impact on motor performance. Damage to higher-order motor areas in the frontal and parietal lobes^43^ that have been implicated in motor planning and execution,^44^ as well as damage to regions important for attention,^45^ may be causally related to chronic motor outcomes. The same rationale underlies the most successful theory-driven models in a previous study.^18^ Further, a patient's ability to recover from or compensate for deficits may depend on a larger extent of lesion damage, the related overall stroke outcome, and related physiological consequences, such as autonomic dysfunction^46^ or inflammatory processes.^47^ Second, there may be features that are not causally related to motor function but are nonetheless associated with long-term motor deficits. An *in silico* study has shown that imaging features with peak anatomo-clinical correlations can be located outside of the true neural correlates of a deficit;^48^ in this specific example, damage in a temporal area correlated highly with a deficit that originated from either inferior parietal or inferior frontal damage. This can be explained by the typical lesion anatomy, which does not damage anatomical structures independently, but in highly systematic patterns imposed by the typical anatomy of the brain vasculature.^49^ Moreover, information from outside critical areas may supplement information in critical areas. For example, an imaging feature within a critical brain region or network may be damaged either by a small lacunar lesion that only causes a minor deficit which can be compensated for or by a large lesion that fully disrupts a functional brain module and causes an irrevocable deficit. Damage to features outside of the critical area that are indicative of a larger lesion might enable differentiation of these cases.

In this paper, structural disconnection of areas outside of the primary motor system was associated with worse motor outcomes. Similarly, the extent of damage to a lesion-behaviour map including voxels that lie mostly outside of the motor system (Supplementary Fig. 9) had a stronger association with motor outcomes than damage to known motor tracts. This study suggests that regardless of whether these extra-primary motor structures are causally related to a deficit, they are more useful biomarkers of chronic motor deficits than the extent of damage to white matter tracts of the motor system.

### Association of lesion-based structural disconnectivity with chronic motor outcomes

Models using ChaCo scores performed best of all models tested, particularly when feature selection was employed. These are high-dimensional models that may require more data to start outperforming simpler models,^22^ which may explain the drop in their relative performance when using smaller subsets of the data for training, including using only chronic data and using only subjects with FMA-UE scores. However, with sufficient data, one strength of models using ChaCo scores can be understood, in part, by considering how lesion data is represented relative to LBM and sLNM models. For LBM and sLNM, the data on which feature selection takes place are voxels. On the other hand, in ChaCo models, the data on which feature selection takes place are regional measures of structural disconnection. This data transformation essentially reduces the number of “rare” features compared to voxelwise representations (Supplementary Fig. 10), as non-overlapping lesions that affect different portions of the same tract are mapped onto ChaCo scores of the same region or set of regions. The drawback to this approach is that regional ChaCo scores do not enable the detection of associations between damage to specific tracts and motor outcomes; if such associations exist then that signal may be diluted in regional measures.

### Feature weights of the ChaCo models

In this paper, our main aim was to compare stroke imaging biomarkers in their association with motor outcome. We did not aim to uncover the precise nature of the neural correlates of motor deficits. However, further investigation of model features and their weights can provide some clarity in understanding the models’ relative performance.

Several grey matter regions that are part of the known motor system were incorporated into ChaCo models with negative weights, suggesting that more damage to these regions is associated with worse motor outcomes. Such regions include the primary somatomotor cortex and subcortical structures, as well as secondary motor structures in the frontal and parietal cortices. Many regions that were consistently assigned negative weights were neighbouring regions, in line with the spatial distribution of motor networks and somatotopy of the motor system. However, several regions, in particular in the right frontal cortex and medial surface, were consistently assigned a positive weight. In other words, some brain regions existed for which feature weights indicated a paradoxical lesion-deficit relationship in the sense that brain damage was linked to a more favourable motor outcome. Some cases of genuine facilitation due to brain damage have been documented,^24,50^ and inhibitory interregional brain modes that can explain paradoxical lesion effects are assumed.^51,52^ Hence, paradoxical lesion effects underlying motor outcome may provide a counter-intuitive, but still viable explanation of our findings. On the other hand, methodological aspects could also be an explanation for apparently paradoxical effects. First, paradoxical associations might arise as an artefact from the lesion anatomy.^53^ For illustration, imagine a stroke population in which some patients suffer from visual field defects after posterior brain damage to the visual system. The existence of a frontal lesion might then be anticorrelated with visual field defects—not because of a true paradoxical lesion effect due to inhibition, but as a mere statistical effect following from the lesion anatomy: a patient with a frontal lesion is unlikely to simultaneously suffer from a posterior lesion and, hence, is unlikely to suffer from visual field defects. Similar effects are imaginable on a smaller scale affecting neighbouring brain regions.^53^

Second, paradoxical effects might also emerge as a simple statistical artefact. The feature weights in a high-dimensional model can be unstable^35^ and, especially with highly correlated data, can somehow be decoupled from causality and the actual structure of the investigated entity. In our study, the stability of features was decent, though still markedly inferior to some previous studies that explicitly optimized feature replicability to create interpretable high-dimensional models.^54,55^ Only for some areas, the paradoxical feature weights were stable across replications. Future studies are needed to validate or optimize our modelling and model interpretation strategies.

### Surprisingly strong performance of a simple biomarker: LBM lesion load

We hypothesized that because of previously identified relationships between structural disconnections and motor deficits, sLNM-LL would outperform LBM-LL. On the contrary, we saw that LBM-LL performed better than sLNM-LL. In some cases, LBM-LL performed as well as complex, high-dimensional ChaCo models. The LBM was derived from an independent dataset, suggesting that this map of association is generalizable to new data. Associating motor deficits with LBM-LL can be done with simple linear regression, making this biomarker accessible to those with a limited coding background. Hence, even though a single lesion load measure might at first glance appear to be overly simple and unfit to represent the complexity of the human brain and its pathology, it might still provide a biomarker that can be meaningful in clinical studies with simple, straightforward interpretable design. However, high-precision personalized medicine should rely on more complex, high-dimensional imaging markers such as ChaCo disconnection scores.

### Ensemble models

Finally, we saw that averaging estimations from multiple models generally improves performance. This suggests that the information captured by each data type is not redundant and that using multiple different lesion metrics may compensate for weaknesses of different feature representations. Beyond estimations of chronic motor scores, models may be improved by testing and possibly combining multiple features as well as multiple feature representations (specifically, LBM-LL and ChaCo scores) to obtain an optimal model.^21,56^

### Limitations

There are several limitations of this study. Without baseline motor scores and baseline lesion masks (which may differ somewhat from the lesion masks collected after the acute post-stroke stage), we cannot evaluate the associative power of baseline lesion damage and baseline behavioural information. Although we lack baseline motor scores, which have traditionally been thought to explain up to 70% of motor recovery after stroke, empirical evidence supporting the hypothesis of proportional recovery has been recently re-analysed.^57,58^ In these analyses, it has been shown that the correlation between baseline motor severity and motor recovery may be inflated by the ceiling effect of several stroke scales. In this sense, baseline imaging may provide useful biological information that is associated with the extent of one's recovery. We hope that our work will inspire further research that includes baseline lesion data, which could validate or refine the relationships we have observed.

Without baseline motor scores, we cannot know to what extent lesion information explains unique variance in chronic motor outcomes compared to baseline motor scores. However, previous studies have shown that models with behavioural features and imaging biomarkers explain more variance in motor outcomes than models with behavioural features alone.^5,10,14^ Similarly, the lack of subject-level rehabilitation data is another limitation. Additionally, the inclusion of metrics that are not specific to motor deficits (i.e. NIHSS) in the training sets may have reduced the performance of models.

Furthermore, ChaCo scores were calculated using a database of healthy young control subjects (aged 26–36) which does not fully reflect the range of ages of the stroke subjects analysed here. However, previous work has shown that structural disconnection estimates based on a young adult white matter tractography atlas are very similar to the same metrics derived from a healthy ageing adults’ white matter tractography atlas.^59^

The associations between lesion volume and motor outcomes have been weak or inconclusive; in particular, the topology of the lesion is more strongly associated with motor outcomes than lesion volume.^10,17,60^ As such, lesion volume was not included as a theory-based biomarker.

Finally, the strength of LBM-LL/sLNM-LL models relative to ChaCo models may be reduced because of the distribution shift in the training versus testing dataset: the sample used to generate the LBM was different from the sample on which it was tested, whereas, for ChaCo models, feature selection was performed using the same dataset on which the models were tested.

## Supplementary Material

fcae254_Supplementary_Data

## Data Availability

To protect the privacy of research participants, individual subject data used in this study are not available in a public repository. Participating research cohorts vary in public data-sharing restrictions as determined by the following: (1) ethical review board and consent documents; (2) national and transnational sharing laws; and (3) institutional processes that may require signed data transfer agreements for limited, predefined data use. However, data sharing is possible for new and participating ENIGMA Stroke Recovery Working Group members who agree to the consortium's ethical standards for data use and upon the submission of a secondary analysis plan for group review. Upon the approval of the proposed analysis plan, access to relevant data is provided contingent on local principal investigator approval, data availability, and compliance with supervening regulatory boards. Deidentified summary data used for this study can be made available upon reasonable request to the corresponding author.
