# Supplementary material for: Data-driven biomarkers better associate with stroke motor outcomes than theory-based biomarkers
Source: Brain Commun. 2024 Jul 31;6(4):fcae254. doi: 10.1093/braincomms/fcae254 (PMC11336660; doi:10.1093/braincomms/fcae254)
Supplement: fcae254_Supplementary_Data [file fcae254_supplementary_data.pdf]

Supplementary Materials

Supplementary Table 1:**Demographic information of subjects with acute or subacute stroke (N=327) in the ENIGMA dataset, by site.** Some sites have both acute and chronic subjects that are listed separately. Total sample size (N), number of females (F) and males (M), and information about age (years), normalised motor scores, time since stroke at the time of assessment (months), and lesion volume (cm^3). IQR, interquartile range.

Site ID	Total N. N (F/M)	Median age in years (IQR)	Median motor score (IQR)	Median time since stroke in mos. (IQR)	Median lesion vol. in cm^3 (IQR)
r005	1 (0/1)	50.0 (0.0)	0.29 (0.00)	5.1 (0.0)	1.69 (0.00)
r009	50 (13/37)	70.0 (18.5)	1.00 (0.05)	0.2 (0.1)	1.65 (6.96)
r025	9 (4/5)	70.0 (19.0)	1.00 (0.09)	3.0 (1.0)	0.52 (1.44)
r028	1 (0/1)	63.0 (0.0)	0.74 (0.00)	5.5 (0.0)	23.87 (0.00)
r031	36 (10/26)	58.5 (13.2)	0.52 (0.38)	4.5 (1.7)	10.83 (38.77)
r038	72 (30/42)	66.5 (21.2)	0.78 (0.66)	2.9 (2.3)	11.53 (41.64)
r040	57 (32/25)	64.0 (22.0)	0.40 (0.50)	1.8 (1.5)	14.72 (58.63)
r047	2 (1/1)	71.0 (2.0)	0.59 (0.26)	4.4 (0.2)	23.78 (19.19)
r049	21 (12/9)	65.0 (16.0)	0.95 (0.00)	0.0 (0.0)	1.30 (2.18)
r050	14 (7/7)	68.0 (16.8)	0.92 (0.10)	0.0 (0.0)	0.33 (0.40)
r053	52 (20/32)	63.5 (20.5)	0.92 (0.17)	3.0 (3.0)	13.50 (28.27)
r054	12 (5/7)	65.5 (11.8)	0.67 (0.83)	0.4 (0.2)	4.06 (14.15)

Supplementary Table 2: Number of subjects with data for each type of assessment, broken down by chronicity (acute-subacute/chronic) and site. FMA UE = Fugl Meyer Assessment of Upper Extremity, Barthel = Barthel Index, NIHSS = National Institutes of Health Stroke Score.

**Acute/subacute**					
**Site ID**	**Fugl Meyer UE**	**Barthel**	**NIHSS**	**Grip Strength**	**Not specified**
r005	1	0	0	0	0
r009	1	0	49	0	0
r025	0	0	0	9	0
r028	1	0	0	0	0
r031	36	0	0	0	0
r038	0	72	0	0	0
r040	0	57	0	0	0
r047	2	0	0	0	0
r049	0	0	21	0	0
r050	0	0	14	0	0
r053	0	0	52	0	0
r054	12	0	0	0	0
**Chronic**					
**Site ID**	**Fugl Meyer UE**	**Barthel**	**NIHSS**	**Grip Strength**	**Not specified**
r001	39	0	0	0	0
r002	12	0	0	0	0
r003	15	0	0	0	0
r004	19	0	0	0	0
r005	27	0	0	0	0
r009	0	0	0	0	60
r025	0	0	0	16	0
r027	28	0	0	0	0
r028	21	0	0	0	0
r031	1	0	0	0	0
r034	15	0	0	0	0
r035	15	0	0	0	0
r038	0	18	0	0	0
r040	0	14	0	0	0
r042	22	0	0	0	0

**Acute/subacute**					
r044	4	0	0	0	
r045	4	0	0	0	
r046	11	0	0	0	
r047	44	0	0	0	
r048	43	0	0	0	
r052	0	0	32	0	
r053	0	0	2	0	

Supplementary Table 3: Test performance of all models evaluated, displaying median R^2 and median correlation of average hold-out performances (i.e. average across 5 outer folds) across 100 permutations. Lighter blue shades represent better performance, and models are listed from theory-based to data-driven. Bemog. = demographics, Ipsi. = Ipsilesional, SMATT LL = sensorimotor tract template lesion load, L/R SMATT LL = left and right sensorimotor tract template lesion load, M1 CST LL = M1 corticospinal tract lesion load, ChaCo = Change in Connectivity, fs86 = FreeSurfer 86-region atlas, feat. select. = feature selection

		Median performance	
Ensemble type	Model name	Correlation	R^2
None	M1 CST-LL	0.395	0.132
	Ipsi. SMATT-LL	0.441	0.175
	L/R SMATT-LL	0.445	0.180
	LBM	0.452	0.200
	sLNM-LL	0.428	0.167
	ChaCo (fs86)	0.458	0.204
	ChaCo (shen268)	0.458	0.200
	ChaCo (fs86) (feat. select.)	0.457	0.202
	ChaCo (shen268) (feat. select.)	0.468	0.212
+ Demog.	M1 CST-LL + demog.	0.424	0.173
	Ipsi. SMATT-LL + demog.	0.457	0.199
	L/R SMATT-LL + demog.	0.462	0.203
	LBM + demog.	0.482	0.207
	sLNM-LL + demog.	0.452	0.191
	ChaCo (fs86) + demog	0.471	0.203
	ChaCo (shen268) + demog	0.468	0.204
	ChaCo (fs86) (feat. select.) + demog	0.471	0.205
	ChaCo (shen268) (feat. select.) + demog	0.481	0.215
ChaCo	M1 CST-LL + ChaCo (fs86subj)	0.461	0.210
	Ipsi. SMATT-LL + ChaCo (fs86subj)	0.478	0.226
	Ipsi. SMATT-LL + ChaCo (shen268)	0.481	0.228
	L/R SMATT-LL + ChaCo (fs86subj)	0.481	0.227
	L/R SMATT-LL + ChaCo (shen268)	0.481	0.228
	LBM + ChaCo (fs86subj)	0.487	0.234
	LBM + ChaCo (shen268)	0.491	0.240
	sLNM-LL + ChaCo (fs86subj)	0.468	0.214
	sLNM-LL + ChaCo (shen268)	0.472	0.217
	M1 CST-LL + ChaCo (fs86subj) (feat. select.)	0.458	0.206
	M1 CST-LL + ChaCo (shen268) (feat. select.)	0.469	0.217
	Ipsi. SMATT-LL + ChaCo (fs86subj) (feat. select.)	0.476	0.223
	Ipsi. SMATT-LL + ChaCo (shen268) (feat. select.)	0.485	0.233
	L/R SMATT-LL + ChaCo (fs86subj) (feat. select.)	0.475	0.222
	L/R SMATT-LL + ChaCo (shen268) (feat. select.)	0.486	0.232
	LBM + ChaCo (fs86subj) (feat. select.)	0.487	0.235
	LBM + ChaCo (shen268) (feat. select.)	0.495	0.243
	sLNM-LL + ChaCo (fs86subj) (feat. select.)	0.462	0.208
	sLNM-LL + ChaCo (shen268) (feat. select.)	0.474	0.222
ChaCo	M1 CST-LL + ChaCo (fs86subj) + demog.	0.476	0.214
+ Lesion load	M1 CST-LL + ChaCo (shen268) + demog.	0.477	0.216
+ Demog.	Ipsi. SMATT-LL + ChaCo (fs86subj) + demog.	0.492	0.230
	Ipsi. SMATT-LL + ChaCo (shen268) + demog.	0.495	0.232
	L/R SMATT-LL + ChaCo (fs86subj) + demog.	0.494	0.231
	L/R SMATT-LL + ChaCo (shen268) + demog.	0.494	0.232
	LBM + ChaCo (fs86subj) + demog.	0.506	0.234
	LBM + ChaCo (shen268) + demog.	0.509	0.237
	sLNM-LL + ChaCo (fs86subj) + demog.	0.484	0.222
	sLNM-LL + ChaCo (shen268) + demog.	0.487	0.224
	M1 CST-LL + ChaCo (fs86subj) (feat. select.) + demog.	0.472	0.212
	M1 CST-LL + ChaCo (shen268) (feat. select.) + demog.	0.482	0.222
	Ipsi. SMATT-LL + ChaCo (fs86subj) (feat. select.) + demog.	0.490	0.228
	Ipsi. SMATT-LL + ChaCo (shen268) (feat. select.) + demog.	0.498	0.237
	L/R SMATT-LL + ChaCo (fs86subj) (feat. select.) + demog.	0.490	0.229
	L/R SMATT-LL + ChaCo (shen268) (feat. select.) + demog.	0.499	0.237
	LBM + ChaCo (fs86subj) (feat. select.) + demog.	0.505	0.234
	LBM + ChaCo (shen268) (feat. select.) + demog.	0.513	0.241
	sLNM-LL + ChaCo (fs86subj) (feat. select.) + demog.	0.479	0.218
	sLNM-LL + ChaCo (shen268) (feat. select.) + demog.	0.492	0.230

Supplementary Table 4: Breakdown of test folds containing entire sites not used in training (note for Fold 1, only sites listed in the same row are not used in training, all other sites are used for training).

Fold	Sites	Total N
1	r028, r042, r034, r046, r004, r003, r045	107
2	r035, r047, r048, r002	114
3	r005, r031, r001, r004, r027	99

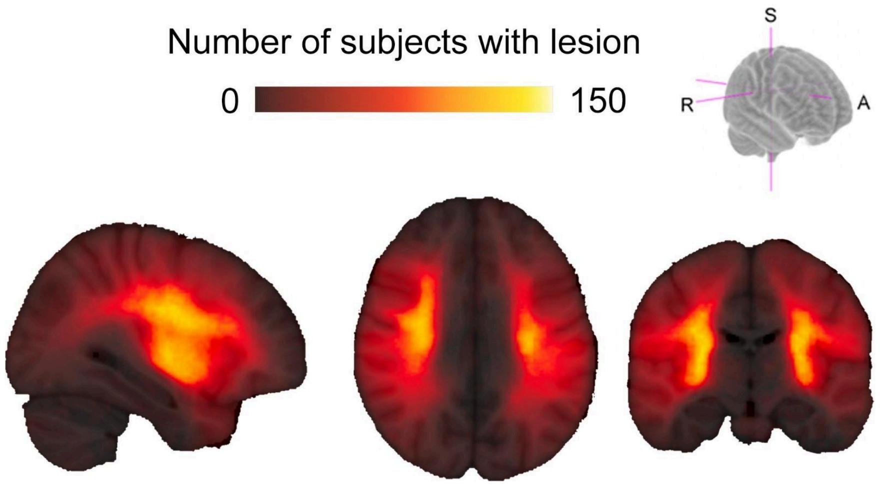

Supplementary Figure 1. Distribution of lesions in the ENIGMA cohort.

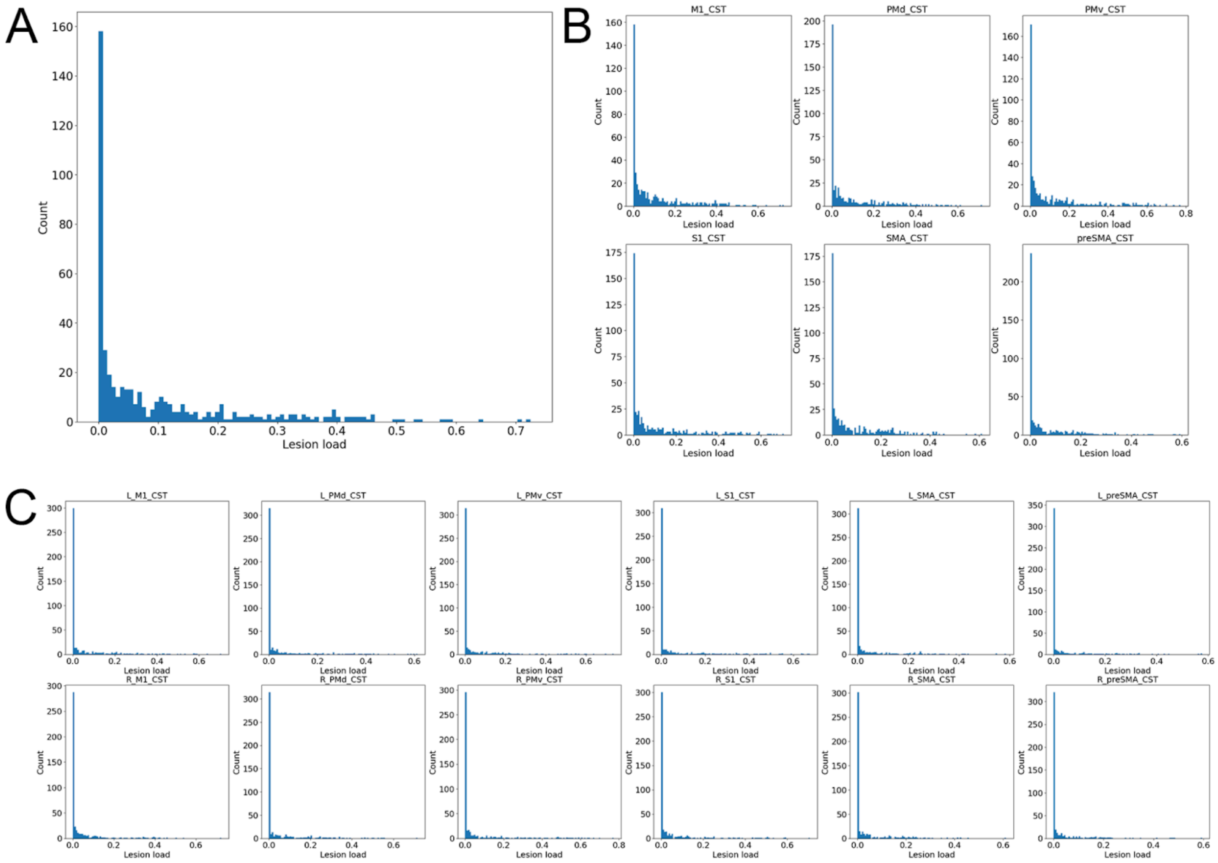

Supplementary Figure 2. Distribution of lesion load variables for chronic subjects. **A**. M1-CST-LL distribution, **B.** Ipsilesional SMATT-LL distribution, **C.** Bilateral SMATT-LL distribution.

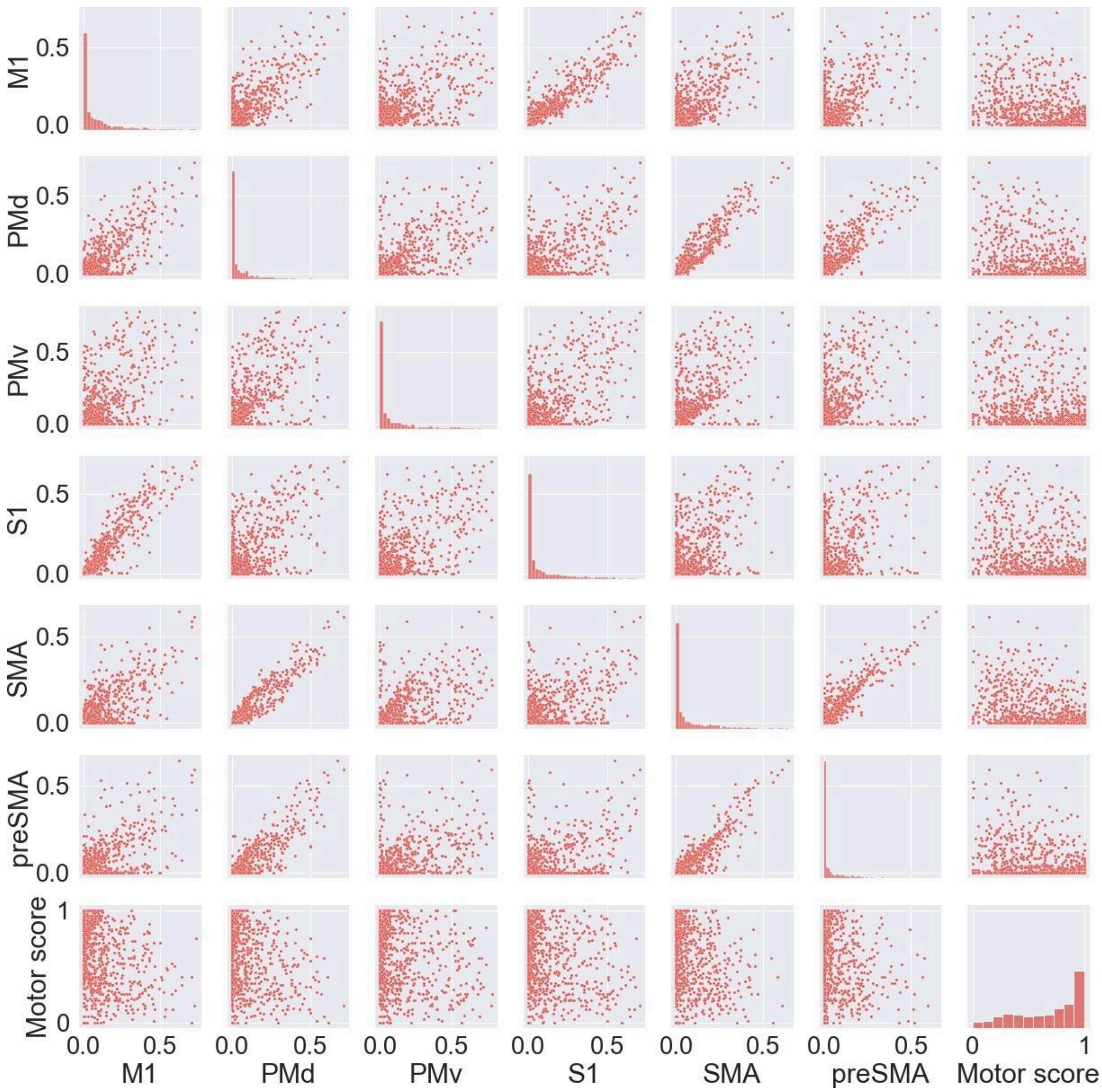

Supplementary Figure 3. Correlations between lesion load calculated for each ipsilesional tract in the sensorimotor area tract template atlas. Acronyms are as follows: primary motor cortex (M1), dorsal and ventral premotor cortex (PMd and PMv, respectively), supplementary motor area (SMA), pre-supplementary motor area (pre-SMA), and primary somatosensory cortex (S1).

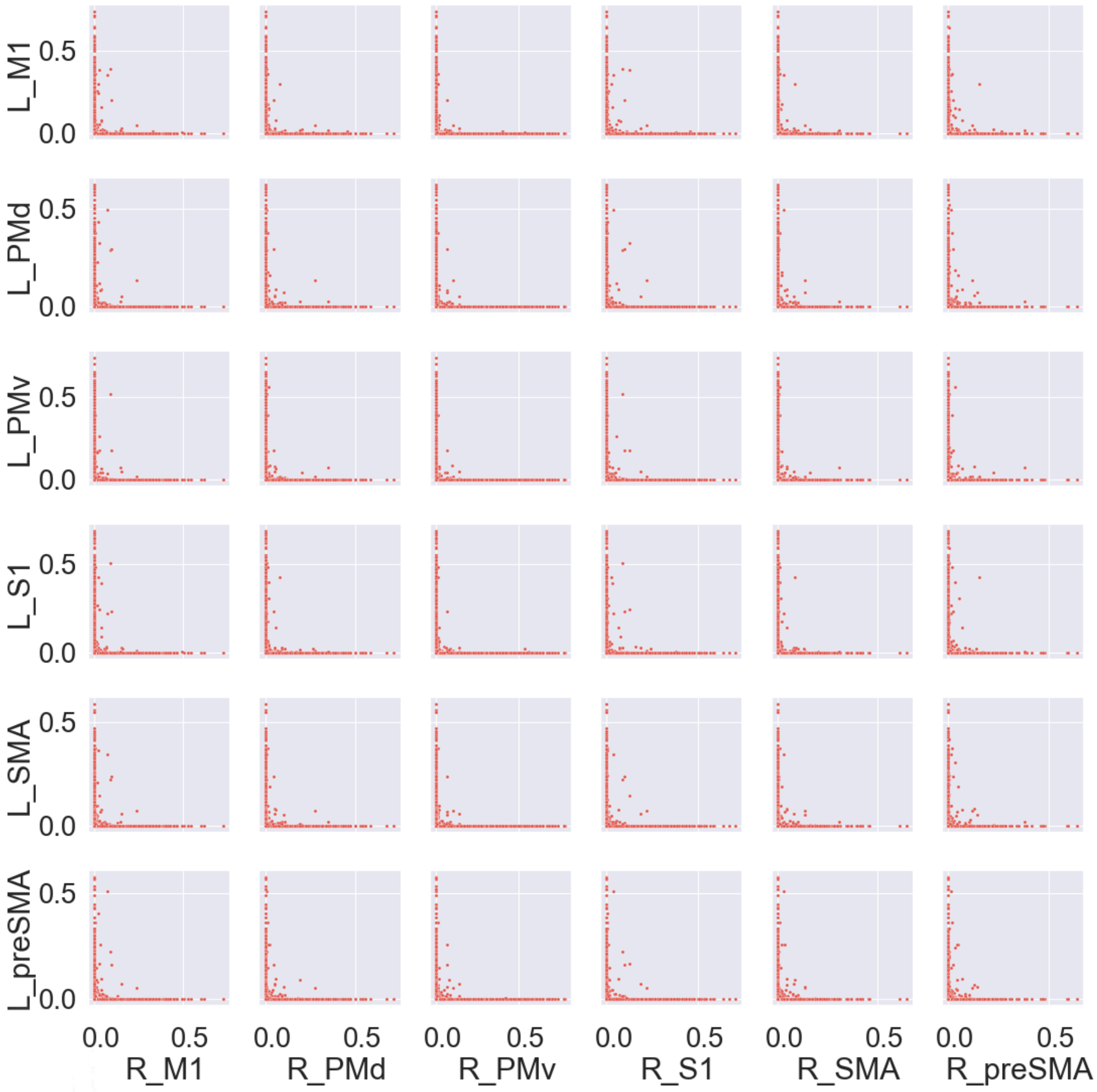

Supplementary Figure 4. Correlations between lesion load calculated for left vs. right hemisphere tracts in the sensorimotor area tract template atlas. Acronyms are as follows: left and right primary motor cortex (M1), dorsal and ventral premotor cortex (PMd and PMv, respectively), supplementary motor area (SMA), pre-supplementary motor area (pre-SMA), and primary somatosensory cortex (S1).

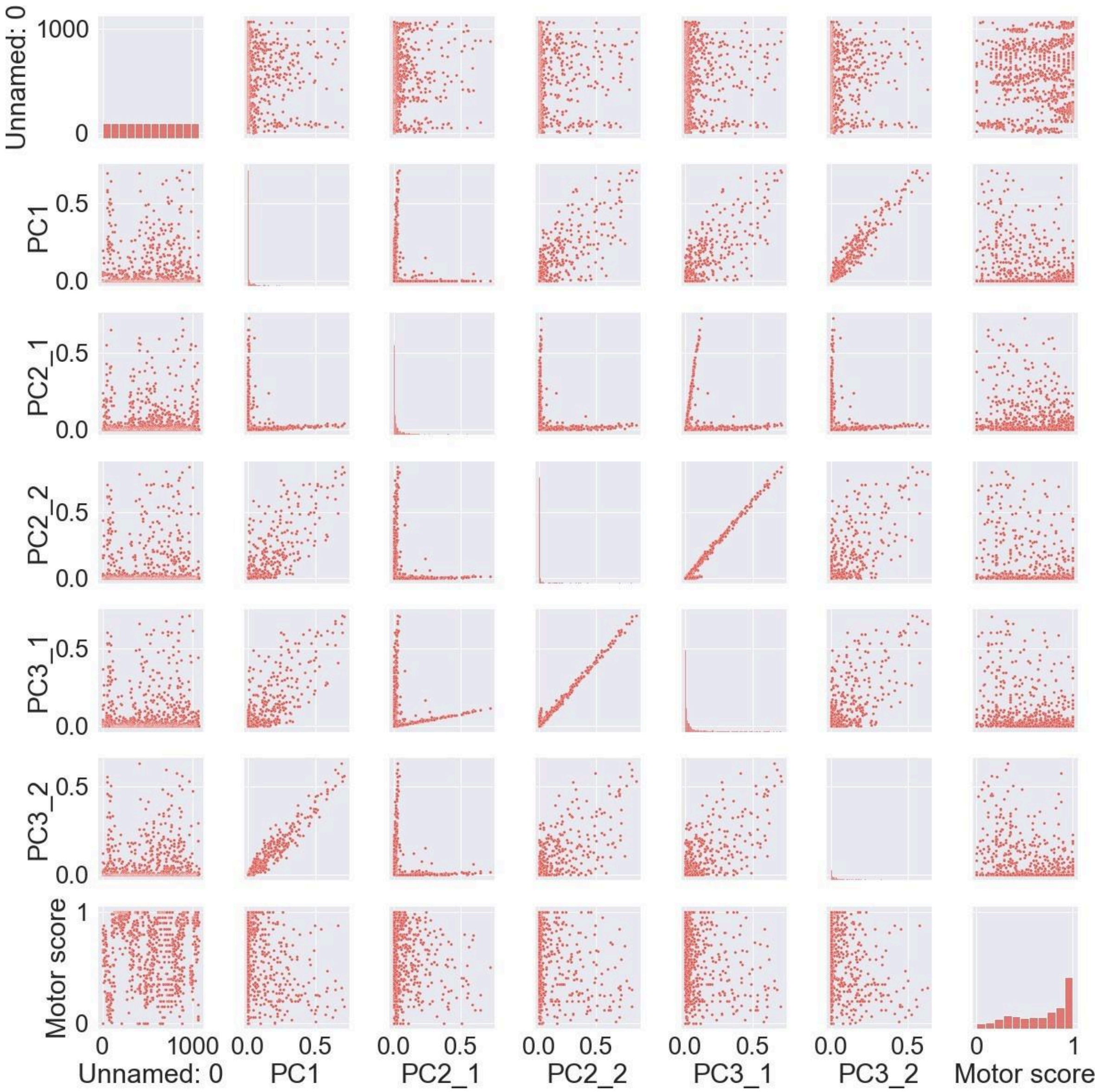

Supplementary Figure 5. Correlations between lesion load calculated for each structural lesion network-derived principal component map. PC = principal component.

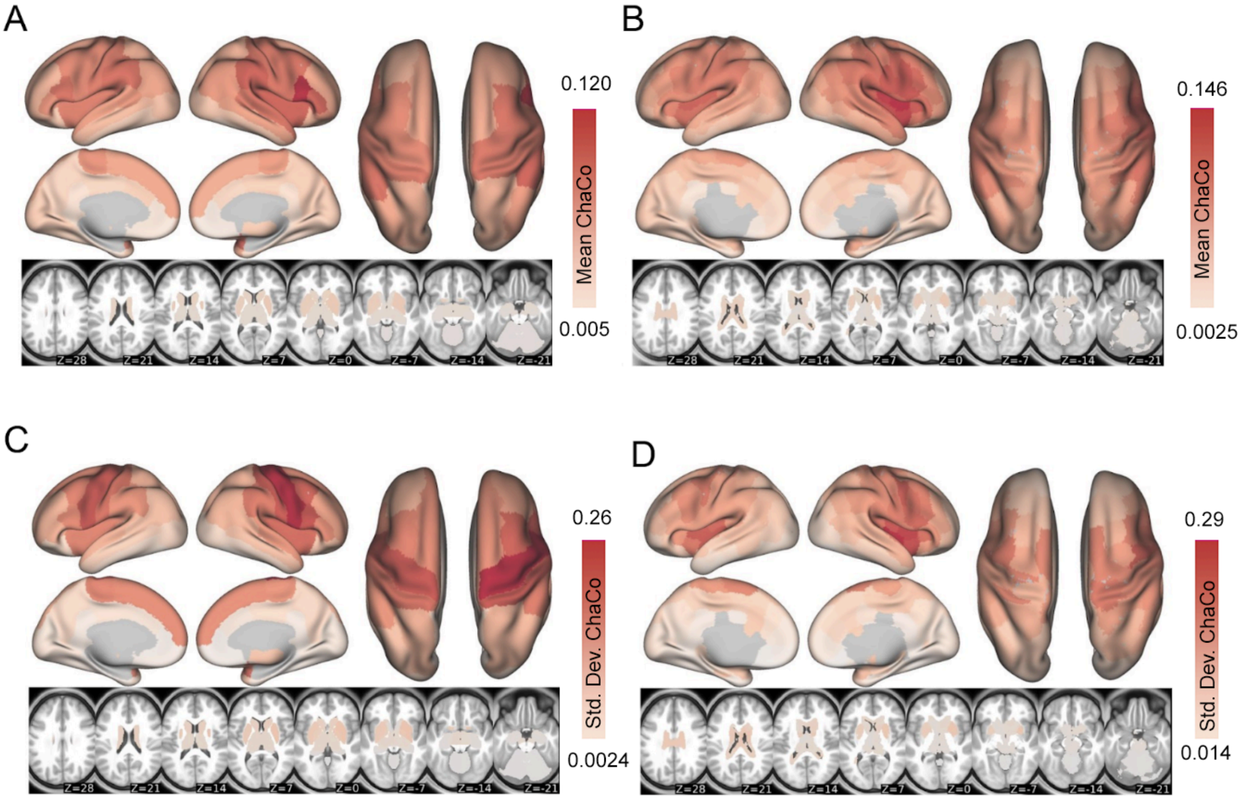

Supplementary Figure 6. Top: Mean regional change in connectivity (ChaCo) scores across acute and chronic subjects used in predictive models. Mean of ChaCo scores parcellated with (**A**) the FreeSurfer 86-region atlas (min. = 0.005, max. = 0.120) and with (**B**) the Shen 268-region atlas (min. = 0.0025, max. = 0.146), normalized to the maximum value across regions (red = higher mean ChaCo score across subjects) Standard deviation of regional change in connectivity (ChaCo) scores across acute and chronic subjects used in predictive models. Standard deviation of ChaCo scores parcellated with (**C**) the FreeSurfer 86-region atlas (min. = 0.024, max. = 0.26) and with (**D**) the Shen 268-region atlas (min. = 0.014, max. = 0.29), normalized to the maximum value across regions (red = higher standard deviation of ChaCo score across subjects)

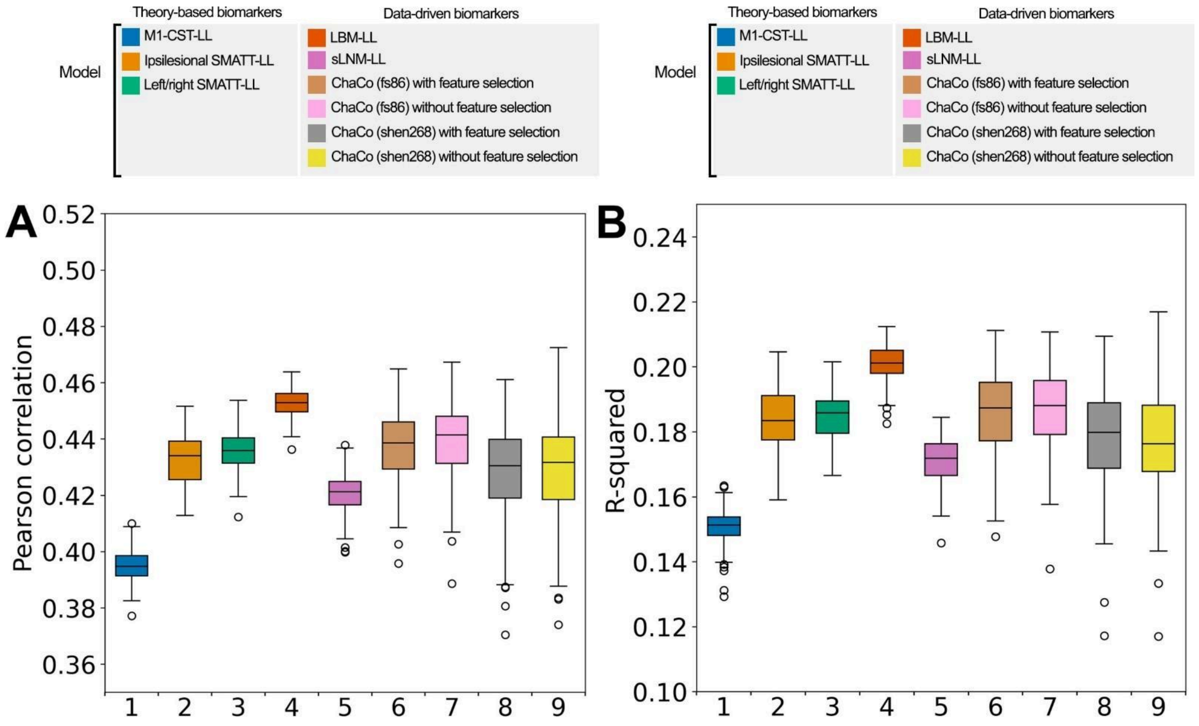

Supplementary Figure 7, Summary of model performance metrics across all models tested using only chronic data for training. **A.** and **B.** Distribution of model performance (mean Pearson correlation/R^2 across 5 outer folds for 100 permutations of the data). Boxplots are colored arbitrarily for clarity. The boxes extend from the lower to upper quartile values of the data, with a line at the median. Whiskers represent the range of the data from [Q1-1.5*IQR, Q3+1.5*IQR]

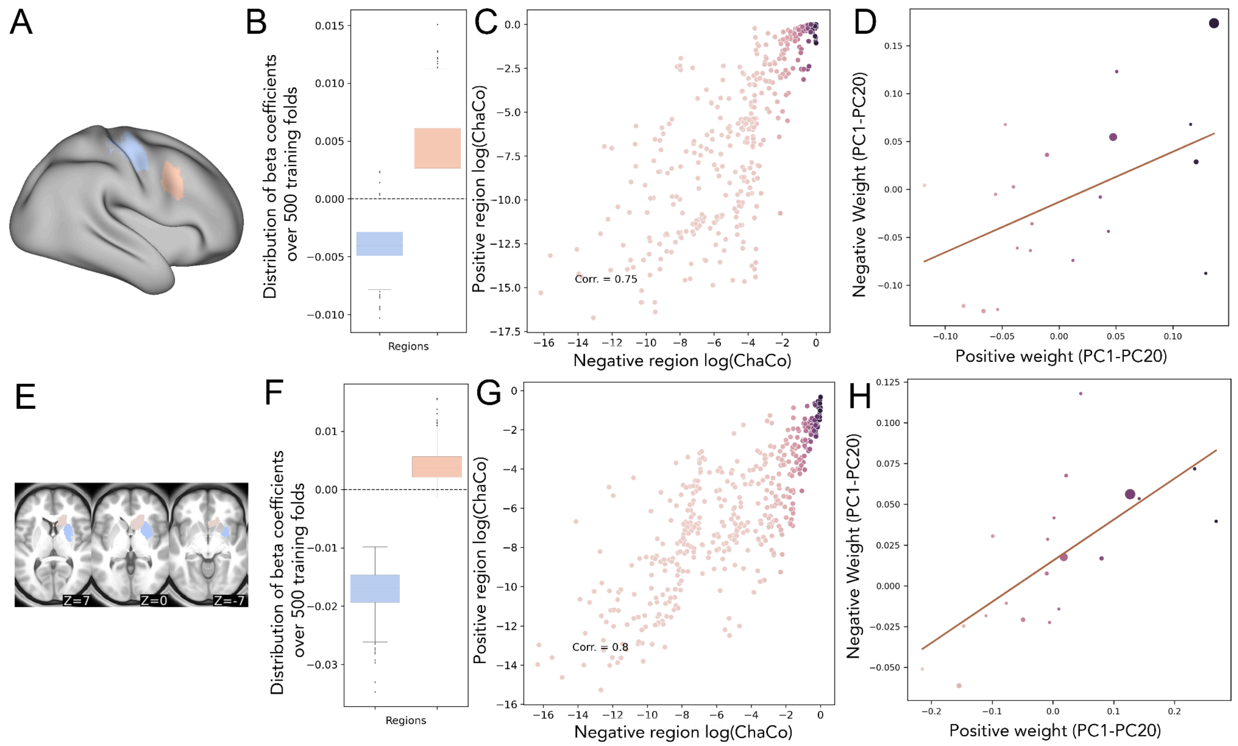

Supplementary Figure 8. The correlation between ChaCo scores for all pairs of consistently-weighted regions was calculated. In total, 4 pairs of regions had ChaCo scores with correlation > 0.8. Two such pairs are plotted in **A** and **E**, coloured based on the median beta coefficient for that region. **B, F.** distribution of beta coefficients for region-pairs with >0.8 correlation in ChaCo scores but opposite sign beta coefficients. **C, G.** Scatterplot showing distribution of ChaCo scores for each region-pair. **D, H.** Region-pairs' loading onto principal components that explain >90% of the variance in 268-region ChaCo score, with the negatively-weighted region on the y-axis and the positively-weighted region on the x-axis. Dots indicate the regions' loading onto each component, and the size of the dots corresponds to the percentage of variance explained in the full dataset by each component.

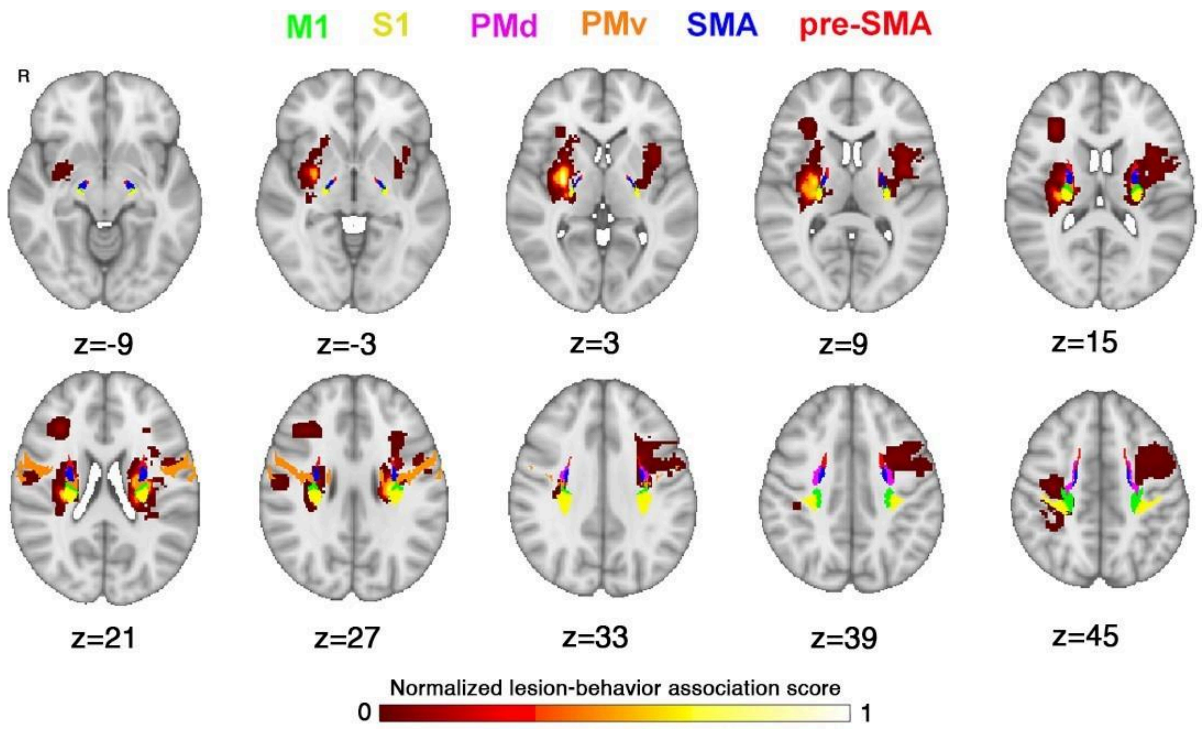

Supplementary Figure 9. Overlap between lesion-behaviour map and SMATT tracts. LBM map is plotted in hot colormap, SMATT tracts are plotted in their original colours (see Fig. 2).

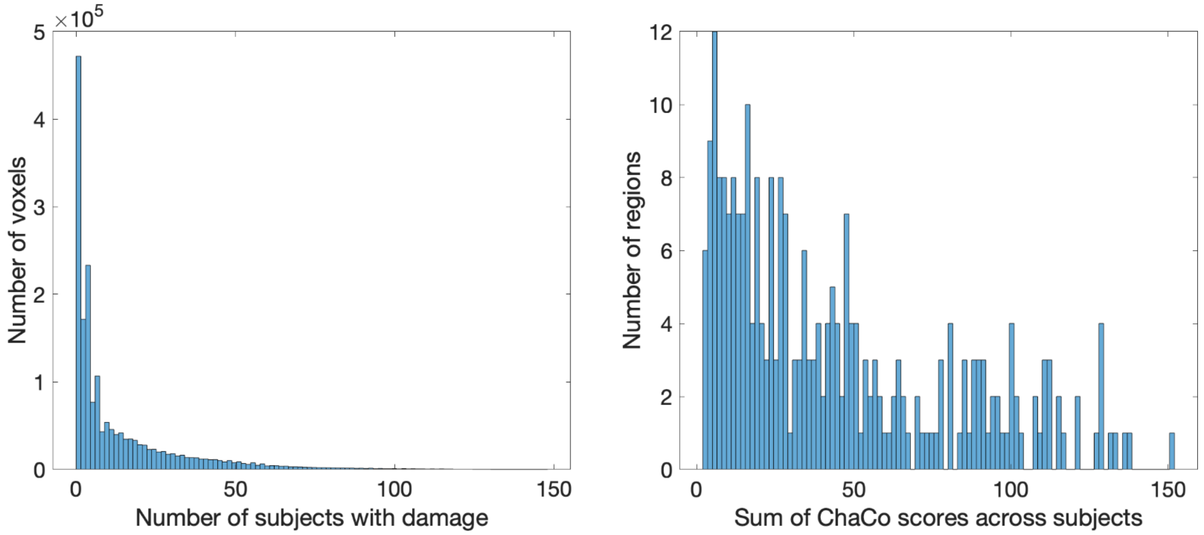

Supplementary Figure 10. Histogram of voxelwise lesion damage and regional (ChaCo scores) lesion damage. Left: Distribution of voxelwise lesion damage within a whole-brain mask. Most voxels are damaged in zero subjects. Right: Distribution of ChaCo scores across all subjects. Every region has some disconnection in at least one subject in the population.
